# Modulating electrophysiology of motor neural networks via optogenetic stimulation during neurogenesis and synaptogenesis

**DOI:** 10.1038/s41598-020-68988-y

**Published:** 2020-07-27

**Authors:** Gelson J. Pagan-Diaz, Jenny Drnevich, Karla P. Ramos-Cruz, Richard Sam, Parijat Sengupta, Rashid Bashir

**Affiliations:** 1 Department of Bioengineering, University of Illinois, Urbana-Champaign, Engineering Hall, 1308 W Green St, Urbana, IL 61801 USA; 2 Nick Holonyak Micro and Nanotechnology Lab, University of Illinois, Urbana-Champaign, Urbana, IL 61801 USA; 3 High Performance Biological Computing and the Carver Biotechnology Center, University of Illinois, Urbana-Champaign, Urbana, IL 61801 USA; 4 School of Molecular and Cellular Biology, University of Illinois, Urbana-Champaign, Urbana, IL 61801 USA; 5 Beckman Institute for Advanced Science and Technology, University of Illinois, Urbana-Champaign, Urbana, IL 61801 USA; 6 Program in Neuroscience, University of Illinois, Urbana-Champaign, Urbana, IL 61801 USA; 7 Present Address: Richard and Loan Hill Department of Bioengineering, University of Illinois, Urbana-Champaign, Chicago, 60607 USA

**Keywords:** Extracellular recording, Optogenetics, Neural circuits, Synaptic plasticity, Biomedical engineering

## Abstract

Control of electrical activity in neural circuits through network training is a grand challenge for biomedicine and engineering applications. Past efforts have not considered evoking long-term changes in firing patterns of in-vitro networks by introducing training regimens with respect to stages of neural development. Here, we used Channelrhodopsin-2 (ChR2) transfected mouse embryonic stem cell (mESC) derived motor neurons to explore short and long-term programming of neural networks by using optical stimulation implemented during neurogenesis and synaptogenesis. Not only did we see a subsequent increase of neurite extensions and synaptophysin clustering, but by using electrophysiological recording with micro electrode arrays (MEA) we also observed changes in signal frequency spectra, increase of network synchrony, coordinated firing of actions potentials, and enhanced evoked response to stimulation during network formation. Our results demonstrate that optogenetic stimulation during neural differentiation can result in permanent changes that extended to the genetic expression of neurons as demonstrated by RNA Sequencing. To our knowledge, this is the first time that a correlation between training regimens during neurogenesis and synaptogenesis and the resulting plastic responses has been shown in-vitro and traced back to changes in gene expression. This work demonstrates new approaches for training of neural circuits whose electrical activity can be modulated and enhanced, which could lead to improvements in neurodegenerative disease research and engineering of in-vitro multi-cellular living systems.

## Introduction

Inducing neuronal plasticity is one of the grand challenges in neuroengineering. Understanding and controlling nerve connectivity and their plasticity could have profound impacts in regenerative medicine^1–4^, as studies have shown that engrafting motor neuron containing embryoid bodies (MEBs) can improve recovery in motor nerve injuries^1,3^. The ability to enhance or control the electrophysiological functions of such MEBs could be used for improvement of motor function recovery. Furthermore, in the emerging field of engineering biohybrid neuronal-driven biological machines, it would be highly advantageous to forward-engineer programmable neural networks that could be installed within in vivo or in vitro systems in order to achieve targeted functional behaviors^5–7^. While the mechanisms responsible for synaptic modulation and circuitry formation (i.e. memory and learning) have been studied in invertebrate and mammals, the complexity of neuronal plasticity pathways, such as potentiation and homeostatic plasticity, have made it difficult to control neural circuit development for in vitro applications^8^. Furthermore, studies have shown varying degrees of emerging phenomena that are correlated to neuronal plastic events that would imply “learning” or “memory storage” in neural circuits^9–13^ These varying degrees extend from neural mechanisms that regulate potentiation to feedback mechanisms with respect to associations to stimulation. Currently, in-vitro micro electrode array (MEA) systems have aimed to study the effects of stimulation protocols in the resulting network dynamics for the past 30 years^13–19^. While some of these studies have induced transient changes in the recorded signals, most have focused on changes of number of action potentials in mature primary neurons and in learning as a result of feedback to strengthen associations to the stimulation patterns. Due to the fact that these phenomena emerge from multiple pathways related to neuronal plasticity, finding new approaches to enhance and modulate these plastic responses in a long-term way would be highly advantageous in the field.

Because most of these studies on plasticity have focused on modulation of mature neurons, we hypothesized that we could induce plasticity-related long-term electrophysiological changes in in-vitro neural networks, by implementing training regimens during early stages of differentiation, i.e. neurogenesis, coupled with training regimens during network formation, i.e. synaptogenesis. To this end, we used 465 nm pulsed light to induce depolarization in specific temporal patterns to implement a training regimen on differentiating channelrhodopsin (ChR2)-expressing MEBs grown in suspension and continued the training regimen after seeding them on functionalized glass to allow for neurite extension and network development. In parallel, we seeded the MEBs on MEA chips while continuing training regimens to be able to characterize the network dynamics during network development. We first characterized responses to the optogenetic stimulation during neurogenesis by assessing morphological parameters, followed by analyzing the resulting electrophysiological responses including the network’s synchronicity, firing patterns in power spectra and the system’s responsivity to stimulation during recording. These results showed a direct correlation between perturbations during differentiation and plastic responses occurring during network formation. Finally, through RNA sequencing studies, we observed genetic changes that serve to explain the observed modulations in these neuronal systems.

## Materials and methods

### Experimental model and subject details

#### mESC culture and differentiation

A feeder layer of mitomycin-C inactivated mouse embryonic fibroblast (MEF) was seeded at a cell density of ~ 3.5 × 10^4^ cells/cm^2^ and cultured in DMEM (Dulbecco’s modified Eagle’s medium) supplemented with 1% fetal bovine serum, 1% L-glutamine and 1% penicillin-streptavidin. Subsequently, HB9:GFP transgenic mouse embryonic stem (mES) cells transfected with (Channelrhodopsin) ChR2-TdTomato were seeded on the feeder layer at a ratio of 1.5 mES cells per MEF. Media was changed to mESC proliferation medium and replaced daily.

Differentiation begun prior to mESC colonies coming in contact with one another. Cultures were trypsinized (0.05% Trypsin) after being exposed to embryonic stem cell differentiation medium (eDM) for an hour, and later seeded on 100 mL low adhesion dishes in 10 mL eDM. The next day, floating cells were collected to separate the culture from adhered non-neuronal lineages. On the following day, embryoid bodies (EBs) were replated in eDM supplemented with 1 µM of retinoic acid (RA) (Sigma Aldrich, MA) and 1 µM puromorphine (PM) (STEMCELL Technologies, MA). On D5, EBs were resuspended in fully supplemented eDM (FS eDM) supplemented with RA and PM plus 10 ng/mL of growth factors, glial cell-derived neurotrophic factor (GDNF) and ciliary neurotrophic factor (CNTF) respectively. Media was changed daily.

#### MEA fabrication

Platinum micro-electrode array chip were fabricated on borofloat glass wafers following standard lithographic techniques. Photoresist LOR3A (MicroChem, MA, USA) was spun on clean substrates, followed by spin coated layer of photoactive S1805 (Dow Chemical Comp., MI). Spun substrates were exposed using an EVG 620 (i-line) aligner (EV Group Inc., Tempe, AZ) and developed. Ti/Pt (1:3) was later evaporated for a total thickness of 1,000 Angstroms. To passivate traces between the detection area and contact pads, 300 nm of silicon nitride was deposited on the entire substrate using Plasma-Enhanced Chemical Vapor Deposition System (PlasmaLab International, WA). Finished chips were diced into 49 × 49 mm squares and fitted with acrylic wells bonded by Dow Corning Sylgard 184 (Ellsworth Adhesives, IL).

To ensure that thermally generated noise voltages were below membrane voltage fluctuations during neuronal electrical activity, electrode impedance was decreased through electrochemical treatment of the electrodes^20^. Platinum black was formed on top of clean electrodes to achieve high surface area using a Gamry Reference 600 Potentiostat. The galvanostatic deposition was achieved by running a chronopotentiometry experiment at 2.83E-6 A/cm^2^ versus Ag/AgCl for 15 s in a solution of dihydrogen hexachloroplatinate (0.08 mM H_2_PtCl_6_-6H_2_O, Sigma Aldrich, with 0.25 g/L of (CH_3_COO)_2_Pb Alfa Aesar) for a total of 21.45 ng (1.71E-2 ng/µm^2^) of crystallized platinum. The impedance reduction due to the platinum black deposition was examined by electrochemical impedance spectroscopy.

#### Electrophysiology recording

MEA measurements were performed using a MEA 2,100-Lite Amplifier (Multi Channel Systems MCS GmbH, Germany) at 37 °C. Electrical activity from ChR2 + MEBs cultured on MEA were measured every other day. Measurements were performed in dark at a sampling rate of 10 kHz for 20 min in FS eDM with sealed covers to keep CO_2_ concentration stable.

Optogenetic stimulation was performed using a laser diode (LD) Driver (Doric Lenses, Quebec, Canada) attached to a single LD, Blue 465 nm with a Fiberoptic Patchcord for an incident intensity of 10 mW/mm^2^ which ensured that the intensity at the samples was still above the 1 mW/mm^2^ limit for ChR2 activation even after refraction from the lid and media. Because embryoid bodies were below 200 µm of diameter, the incident intensity of the blue light could penetrate across the entire EB^21^. Stimulation patterns were designed with Doric Neuroscience Studio. Optogenetic potentiation regimens were performed in a 3D printed casing which held the fiberoptic in place against the dish lid. During neurogenesis, stimulation was achieved by collecting MEBs and resuspending in a 35 mm dish which fit in the 3D printed casing. During recording, stimulation was done by mechanically holding the fiberoptic against the MEA lid. Potentiation regimens consisted of 5 ms pulses at 20 Hz for one second every other second for 1 h for a total of 1,800 cycles, while stimulation during recording consisted of the same pattern for only 10 cycles at equidistant times during the 20 min recording sessions. Measurements and calculations of heat transfer from beam to the media showed that the energy imparted would cause temperature fluctuations of less than 1 °C, which are less than common fluctuations from normal cell handling.

#### Immunostaining and imaging

Immunocytochemistry was performed on samples fixed with 4% paraformaldehyde and treated with 0.05% Triton-X. Permeabilized samples with blocked with 4% bovine serum albumin at 4 °C overnight. Samples were later stained with primary antibodies at 4 °C overnight, followed by staining of secondary antibodies at room temperature for 2 h. All antibodies were diluted in Antibody Diluent (ThermoFisher Scientific, MA). The used antibodies were 1) anti-NeuN antibody for neural populations, (clone A60, Alexa Fluor 555 conjugate), 2) anti-GAD65/67 polyclonal goat antibody (Santa Cruz Biotechnology), 3) anti-vGlut polyclonal guinea pig antibody (Synaptic Systems, Germany), and 4) DAPI as a nuclear stain. For synaptophysin clustering quantification between MEBs, the used primary antibody was anti-synaptophysin 1 polyclonal chicken antibody (Synaptic Systems, Germany). It was counter stained with goat anti-chicken IgY AlexaFluor 647 (Abcam, MA), respectively, along with DAPI as a nuclear stain. After washing overnight at 4 °C in blocking buffer, samples were mounted with Prolong Diamond antifade (ThermoFisher, MA) and imaged using Zeiss 880 Confocal microscope (Carl Zeiss Microscopy).

#### Scanning electron micrographs

SEM images were taken at 1 kV after grounding the entire array through the contact pads, to avoid charging the insulating Nitride layer, at 100-300X magnification.

#### RNA extraction

Samples of MEBs were carefully collected and centrifuged at 14,000 rpm for 15 min. After aspirating supernatant, samples were flash frozen using liquid nitrogen and immediately stored at -80 °C, until mRNA extraction. Total RNA was collected and purified using the RNeasy Mini Kit Part 1 (Qiagen). The total RNA concentration was quantified using a Nanodrop spectrophotometer.

### Quantification and statistical analysis

#### Spike/burst analysis

Multi-Channel Analyzer software was used for counting and analytically extracting temporal parameters of fast events. Raw data was digitally filtered using a 2nd Order Butterworth high pass filter (cutoff frequency: 200 Hz). Action Potentials (APs), were detected as “spikes” by setting a threshold at 5 × standard deviations from the noise magnitude distribution. Analysis of firing rate behaviors and burst parameters followed spike detection^22^. For this experiment, burst detection was defined by the following parameters:

- Max interval to start burst: 50 ms
- Max interval to end burst: 50 ms
- Min. interval between bursts: 100 ms
- Min. duration of burst: 50 ms
- Min. number of spikes in burst: 4

This analysis was done for active electrodes which were defined as electrodes which recorded at least 10 AP/min.

#### Spectral analysis

Spectral analysis was performed on the slow component of raw data to assess modulations in network behavior. Data extracted as ASCII files were filtered in MATLAB using Butterworth 2^nd^ order high pass digital filter with a cutoff frequency of 200 Hz. This filtered data was then used to detect the occurrences of spikes. Spectral components in the frequency domain from this binned spike data were obtained through Fast Fourier transform (FFT) between 0.1 and 200 Hz to remove DC components from data and detect frequency components occurring in bursts or clusters of spikes.

FFT was obtained for subsequent non-overlapping intervals of 10 s across the initial 4 min of spontaneous activity and normalized by the area under the curve. Calculated spectra were smoothed using a 3-point window moving average. Data was stored in 2D matrices, summed across electrodes and averaged across MEAs.

#### Synaptophysin cluster counting

Stack images were superimposed for 10 microns in ImageJ. Then a binary threshold was set so that only saturated pixels were conserved. Images were sampled ten times at regions in between MEBs with a 150-by-150 µm area across three biological repeats by day.

#### Quantifying network synchronicity

Overall network correlation was assessed through the automated use of spike data and a customized MATLAB code that calculated cross-correlation for discrete functions, as follows:

$$\left( {f*g} \right)[n]\,\underline{\underline{{{\text{def}}}}}\, \sum\limits_{{m = - \infty }}^{\infty } {f^{*} } \left[ m \right]g[m + n]$$

Here, f and g denote the discreet functions at point n, which represent the recorded MEA signals. Furthermore, f* denotes the complex conjugate and m is the lag or displacement, meaning a feature in f occurring at n that occurs in g at n + m^23^. Spike train data for each channel was cross-correlated with every other channel. Results were normalized so the autocorrelations have unit value at zero lag. The value at zero lag (t_ch-x_ = t_ch-y_) was stored for each correlation in a 60-by-60 matrix relating every channel to each other and plotted as a heat map and a bar graph was used to plot the average.

#### RNA sequencing analysis

Each sample that was categorized as an N for the RNA Sequencing consisted of an entire culture of mESC grown and differentiated from a 35-mm well which resulted in around 100–150 MEBs. Biological triplicates were obtained by thawing and evenly dividing three different vials of 2E6 mESC (< P7) evenly across three wells for a total of 9 seeded wells, and each was handled independently. After expansion and neural induction steps (at D2), one well pertaining to each of the 3 thawed vials were snap frozen. Furthermore, one well pertaining to each of the 3 thawed vials were assigned to be trained, and the remaining three served as controls. At D9, all MEBs were collected in their separate samples and snap frozen for RNA extraction and downstream RNA Sequencing.

The RNA-seq libraries were prepared with Illumina's 'TruSeq Stranded mRNA-seq Sample Prep kit' (Illumina). The libraries were quantitated by qPCR and sequenced on one lane on a HiSeq 4,000 for 101 cycles from one end of the fragments using a HiSeq 4,000 sequencing kit version 1. Fastq files were generated and demultiplexed with the bcl2fastq v2.20 Conversion Software (Illumina). Adapters and low-quality bases were trimmed from reads using Trimmomatic^24^ (v0.36) with parameters LEADING:28 TRAILING:28 MINLEN:30. The trimmed reads were quasi-mapped to Gencode’s M19 transcriptome using Salmon^25^ (v 0.8.2) with additional parameters –seqBias –gcBias –numBootstraps = 30. Transcript expression value were summarized to the gene-level and corrected for average transcript length using the “lengthScaledTPM” method^26^. TMM-normalized^27^ log2 counts per million (cpm) values (prior.count = 3) were calculated and only genes with > log2(0.5 cpm) in at least 3 samples were analyzed using the limma-trend method^28^. Three pairwise comparisons were made (C vs D2, S vs D2 and S vs C) and multiple testing correction was done separately for each comparison using the False Discovery Rate method^29^; significant differential expression was indicated at FDR p-value < 0.05.

#### Statistical analysis

All data sets from electrophysiological responses were extracted from raw recordings using Matlab (Mathworks, Natick MA, USA). To validate the MEB-derived neural networks by measuring the response to known signaling molecules, one MEA per signaling molecule was used, selecting 15 active electrodes. Testing responses of neurite extension to training regimens during neurogenesis was done by monitoring and imaging 20 separate embryoid bodies seeded on gridded coverslip and measuring and averaging at the 4 cardinal points for each measured embryoid body. Response of presynaptic puncta to stimulation regimens was measured for 10 images from 40x  objectives of separate cultures. Electrophysiological responses were measured for three cultures grown on separate MEAs per experimental group, with each replicate value obtained from averaging active electrodes from each culture. All results, unless stated otherwise, are expressed as mean + /- SEM. Statistical comparisons were performed in OriginLab using one-way ANOVA and repeated measures ANOVA (when appropriate) for group and longitudinal difference test and followed by post-hoc Tukey test for multiple comparisons when applicable. Differences were considered statistically significant if p < 0.05.

## Results

**Figure 1:**
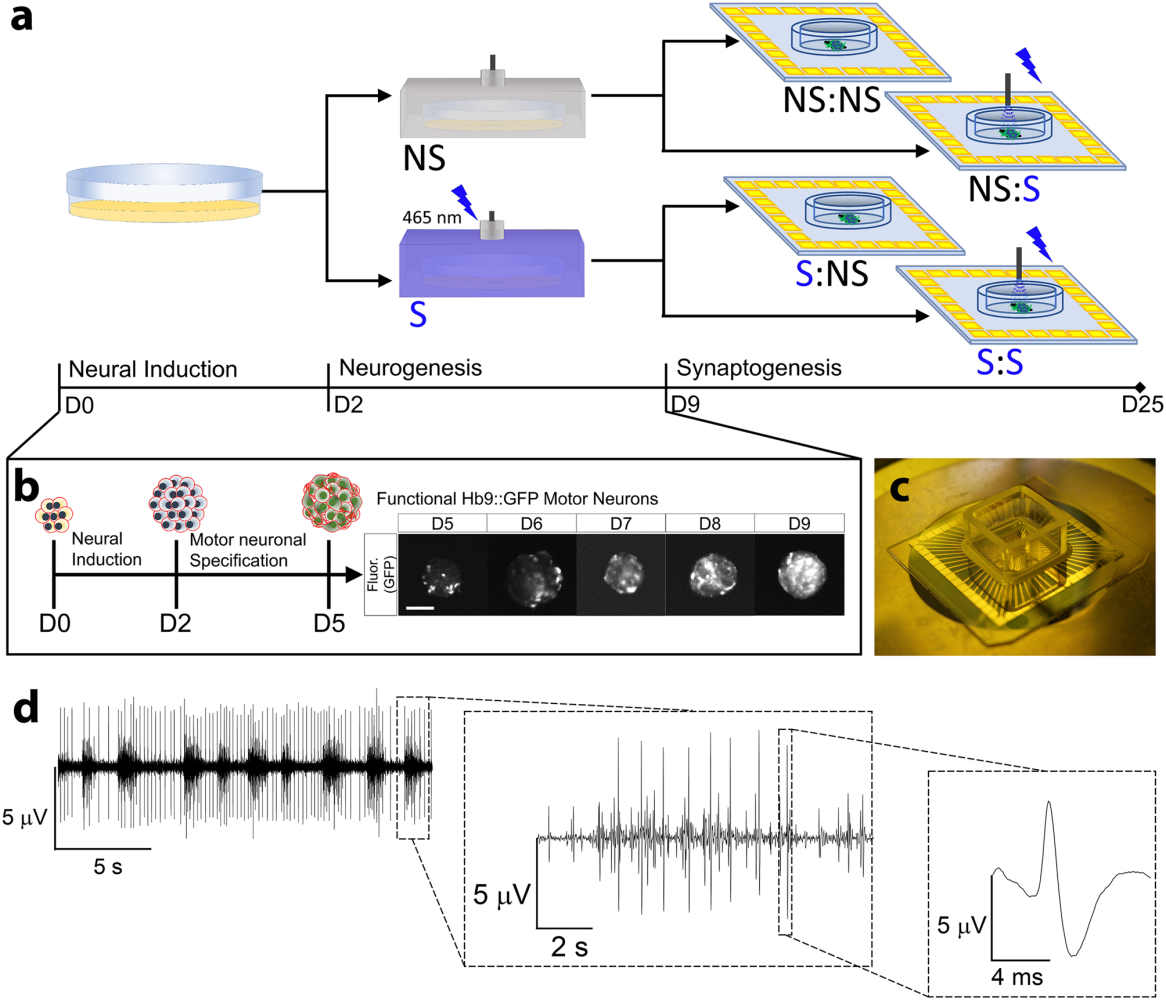
Approach to training mESC-derived motor neuronal embryoid body networks during neurogenesis and synaptogenesis. **a** Representative diagram of experimental setup combining differentiating ChR2 mESC’s and MEAs. **b** Representative diagram of ChR2 mESC differentiation toward motor neuronal embryoid bodies monitored by the expression of GFP guided by the motor neuronal specific Hb9 promoter (scale bar: 200 µm). **c** Representative image of fabricated MEA chip. **d** Representative spontaneous spike trains from MEA recordings of cultured embryoid body networks.

Optogenetic stimulation was used on mESC-derived MEBs to implement training regimens during two important stages of neural development: neurogenesis (while still in suspension) and synaptogenesis (seeded on functionalized glass or MEAs) (Fig. 1a). Training regimens consisted of periodic stimulation with 5 ms pulses at 20 Hz in 1 s intervals for an hour (Supplementary Fig. 1a). This regimen has been shown to enhance axonal growth^30^, and thus would suggest that it could lead to a shift in structural potentiation in a neural network. The regimen was repeated every 24 h as differentiation occurred within the EBs, with an expectation that consistent repetition would enhance the potentiation and cause long-term changes in the firing patterns of the network. Following established differentiation protocols of mESC towards mature motor neurons^31–33^, the described training regimen was started at D2 of differentiation, at which point stem cells have been induced towards neuronal lineages, and specialization and maturation of motor neurons has been shown to take place in the subsequent 7 days (Fig. 1b). Since one of the transcription factors that drove differentiation, retinoic acid, is light sensitive, media was changed every single day immediately after stimulation to ensure that stimulation effects on MEBs were not artifacts (i.e. false positives) caused by photodegradation of factors (Supplementary Fig. 1b)^34^. Furthermore, since the differentiation was monitored with the expression of the motor neuronal marker Hb9 through a GFP reporter, we used the plateau of GFP expression between D8 and D9, as an indicator that D9 was an appropriate time point for seeding the MEBs on glass (Supplementary Fig. 1c). Thus, after these 7 days (D2-D9) of differentiation, stimulated (S) and non-stimulated (NS) cultures were seeded on MEA chips (Fig. 1c). Careful seeding practices were applied to ensure that ~ 20 MEBs were seeded within the sensing area of the MEAs for a ~ 50% coverage by the MEBs (Supplementary Fig. 2). Seeding in this manner ensured empty space between clusters for the extension of processes, even though some nearby clusters would start fusing into larger clusters. The resulting two groups of samples seeded on MEAs were further subdivided into two more experimental groups, referring to whether or not a training regimen was continued during network formation on chip for the consequent 15 days (D10-D25). For ease of discussion, S or NS prior to a colon (e.g. S:X or NS:X) will refer to the presence or lack thereof of stimulation, during neurogenesis, while S or NS written after a colon (e.g. X:S or X:NS), indicates the presence or absence of stimulation during synaptogenesis (Fig. 1a).

**Figure 2:**
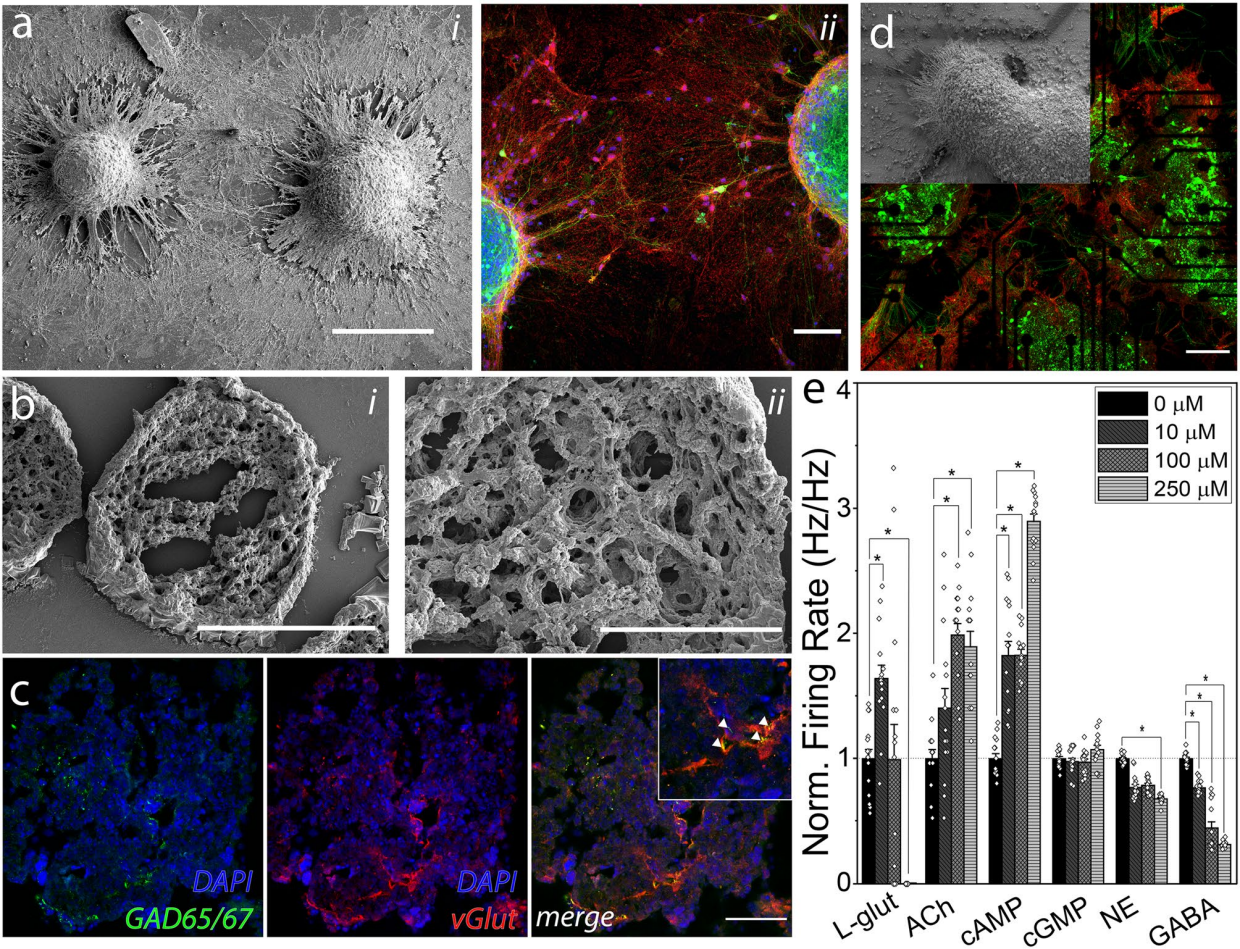
Intact MEBs indicate formation of internal networks and form active networks between them **a** (i) Scanning electron micrograph of two embryoid bodies. (scale bar: 200 µm) and (ii) confocal image showing dense clusters of synaptophysin between cultured embryoid bodies (scale bar: 50 µm). **b** (i) MEB cryosections showing usual internal structure. (Scale bar: 50 µm) with (ii) zoom in of internal structure of a sectioned embryoid body (scale bar: 15 µm). **c** Representative confocal image of MEB cryosection stained for GAD65/67 and vGlut. Triangles show GAD65/67 clusters **d**. Representative confocal image of entire field of view for neural culture grown on the MEA sensing area (scale bar: 200 µm) with scanning electron micrograph zoom in of embryoid bodies extending processes atop of sensing electrodes. **e**. Bar graph for average firing rate of 15 active electrodes for cultured embryoid body networks exposed to known neuronal signaling molecules at sequential addition of tonic baths of 10, 100 and 250 µM. *Glut* Glutamate, *ACh* Acetylcholine, *cAMP* cyclic AMP, *cGMP* cyclic GMP, *NE* norepinephrine, *GABA* gamma-aminobutyric acid) across 5 min of recording/exposure (n = 15; error bar represents SEM, * p < 0.05; ANOVA with Tukey post-hoc test).

The electrical activity of the resulting neuronal cultures was measured with the MEA system and the raw data was filtered to remove low frequencies (< 200 Hz), to remove undesired voltage artifacts (e.g. stimulation artifacts), and extract action potentials recorded as spiking events (Fig. 1d). A two-step procedure was used to remove false positives from the analyzed data: (1) the detection threshold was set at a value at which no positives would be detected from the ground electrode, then (2) the recorded spikes at each electrode were inspected to ensure that the detected spikes had the appropriate voltage phases relating to action potentials: depolarization, repolarization and refractory period.

### MEB cultures form active neural networks with excitatory and inhibitory populations

In this work, neural networks were cultured from intact MEBs, in contrast to growing them as a monolayer after dissociation. The long-term goal of our study is the modulation of electrical activity of the MEBs towards downstream implantation in in-vivo or in-vitro experimental systems and modulating the functionality of such systems through the resulting interaction. When cultured in their intact form, MEBs tend to keep their spheroid shape, while extending processes which contain neurites that form networks as they undergo synaptogenesis (Fig. 2a). Furthermore, dense web-like neurite structures form within the spheroid itself (Fig. 2b) and both excitatory (vGlut) and inhibitory (GAD65/67) receptors stain positively (Fig. 2c).

Network formation was validated by exposing MEB cultures grown on MEAs (Fig. 2d) to varying concentrations of commonly used exciting and inhibiting signaling molecules for 5 min: L-glutamate, acetylcholine, cyclic AMP, cyclic GMP, norepinephrine and GABA. (Fig. 2e). As expected, L-glutamate evoked a statistically significant (repeated measures ANOVA with a Greenhouse–Geisser correction, n = 15; F(1.28,17.89) = 18.78, p = 1.88E-4) response in the network. A post hoc Tukey test showed a statistically significant positive difference at p < 0.05 between 0 µM to 10 µM, while higher concentrations, 100 µM and 250 µM, showed a decrease in firing rate with the latter showing a statistically significant negative difference to the spontaneous firing rate, most likely related to excitotoxicity^35^. Other excitatory signaling molecules, acetylcholine and cyclic AMP, evoked a continuously excitatory response (repeated measures ANOVA; ACh (with Greenhouse–Geisser correction), n = 15: F(2.13,29.78) = 16.14, p = 1.31E-5 and cAMP: F(3,42) = 125.49,p = 4.20E-15) continued a gradual increase in firing rate with increasing concentrations. Cyclic GMP, another cyclic nucleotide similar in function as cAMP, failed to evoke any statistically significant effect on firing rate (repeated measures ANOVA with a Greenhouse–Geisser correction, n = 15; F(2.08,29.18) = 2.86, p = 0.07). On the other hand, the inhibitory neurotransmitters evoked statistically significant effects on the MEB-derived networks, with norepinephrine (repeated measures ANOVA, n = 15; F(3,42) = 81.43, p = 1.53E-17), showing a statistically significant decrease at p < 0.05 in a post hoc Tukey test from 0 µM to 10 µM, and 100 µM to 250 µM, while GABA (repeated measures ANOVA, n = 15; F(3,42) = 191.55, p = 1.60E-24) showed a statistically significant decrease in firing rate at p < 0.05 in post hoc Tukey test at each concentration. The responses corroborated the development of endogenously active neural networks expressing different kinds of receptors. The observations that MEBs extend processes within the body itself while responding to both excitatory and inhibitory signaling molecules would lead to the hypothesis that these MEBs could be forming intrabody circuits which could be “trained” during differentiation and have these changes last after network formation.

#### Stimulation during neurogenesis results in morphological changes in MEB cultures

**Figure 3:**
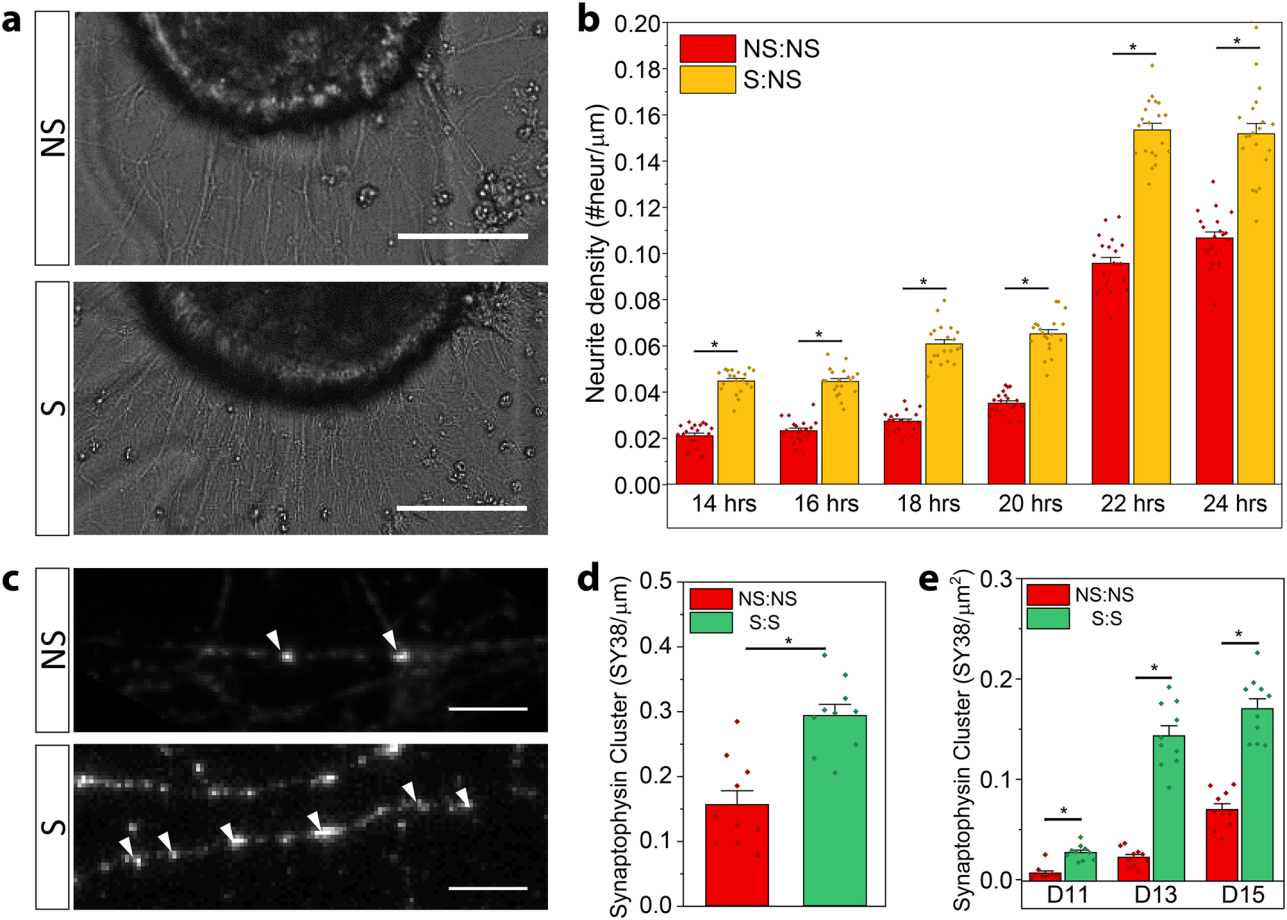
Stimulation during neurogenesis affects key morphological parameters of network formation. **a.** Representative phase contrast images of neurite extension along the periphery of embryoid bodies between non-stimulated (NS) and stimulated during neurogenesis (S) samples (scale bar: 50 µm). **b.** Bar graphs representing the average number of neurites protruding from the periphery of embryoid body normalized by the perimeter of the embryoid body at a given time after seeding. Each point signifies the number of extending neurites normalized by the perimeter of an individual embryoid body (n = 20; error bar represents SEM, *p < 0.05, ANOVA with Tukey post-hoc test). **c.** Representative fluorescence images of synaptic puncta stained against SY38 at D11 along a neurite. Arrow denote presynaptic puncta. (scale bar: 5 µm). **d.** Bar graphs representing the average number of presynaptic puncta along the length of neurites for D11. Each point corresponds to the average number of synaptic puncta along a neurite normalized the length of the neurite per field of view (n = 10; error bar represents SEM, *p < 0.05, ANOVA with Tukey post-hoc test). **e.** Bar graphs representing the average number of presynaptic puncta per unit area for D11-D15. Each point corresponds to the average number of synaptic puncta per unit area in an individual field of view (n = 10; error bar represents SEM, *p < 0.05, ANOVA with Tukey post-hoc test).

The effects of stimulation during differentiation were initially observed in neurite extension and presynaptic protein clustering. While it has been reported that neurite outgrowth could be enhanced if neural populations simultaneously underwent optogenetic stimulation^30^, it was not clear if effects of the stimulation on MEBs done in suspension would still result in an increase of neurite extension when later seeded on chips, as this would indicate some stable long-term changes in the neuronal system. To quantify this, S:NS and NS:NS MEBs were seeded at low confluence on gridded coverslips and imaged 6 times every two hours on D10 (1 DIV) to quantify the number of extending neurites (Fig. 3a). Observations showed a consistently statistically significant positive difference (ANOVA, n = 20; 14hrs: F(1,38) = 215.44, p = 0.0; 16hrs: F(1,38) = 148.40, p = 1.08E-2; 18hrs: F(1,38) = 257.32, p = 0.0; 20hrs: F(1,38) = 199.14,p = 1.11E-2; 22hrs: F(1,38) = 221.35, p = 0.0; 24hrs: F(1,38) = 76.11,p = 1.31E-2) of number of neurites extended for S:NS samples, compared to NS:NS, for each of the six hours the two groups were measured and compared. This indicates an increased rate of neurite extension as a result of the stimulation during neurogenesis (Fig. 3b). Next, we wanted to observe the effect of stimulation during differentiation on the propensity of the network to form synapses. To quantify this, the clustering of presynaptic synaptophysin stained with anti-SY38, was counted along individual neurites as well as per unit area between the groups NS:NS and S:S (Fig. 3c). By D11 (2 DIV) S:S samples showed a statistically significant ~ twofold increase (ANOVA, n = 10; F(1,18) = 24.58, p = 1.02E-4) of synaptophysin clusters per neurite than NS:NS samples (Fig. 3d). This increase of pre-synaptic clusters per neurite combined with the increase in neurite extension resulted in S:S samples presenting a statistically significant higher synaptophysin clusters per unit area than NS:NS counterparts at D11 (ANOVA, n = 10; F(1,18) = 40.18, p = 5.68), D13 (ANOVA, n = 10; F(1,18) = 131.58, p = 1.04E-9) and D15 (ANOVA, n = 10; F(1,18) = 74.87, p = 7.88E-8) (Fig. 3e). When monitoring the difference of pre-synaptic clusters per unit area at D13 and D15, the statistically significant difference indicated that optogenetic stimulation during neurogenesis evoked physiological responses on two important aspects of neural network development: neurite extension and presynaptic clustering (Fig. 3e).

#### MEB network synchronicity is amplified by stimulation during neurogenesis and synaptogenesis

**Figure 4:**
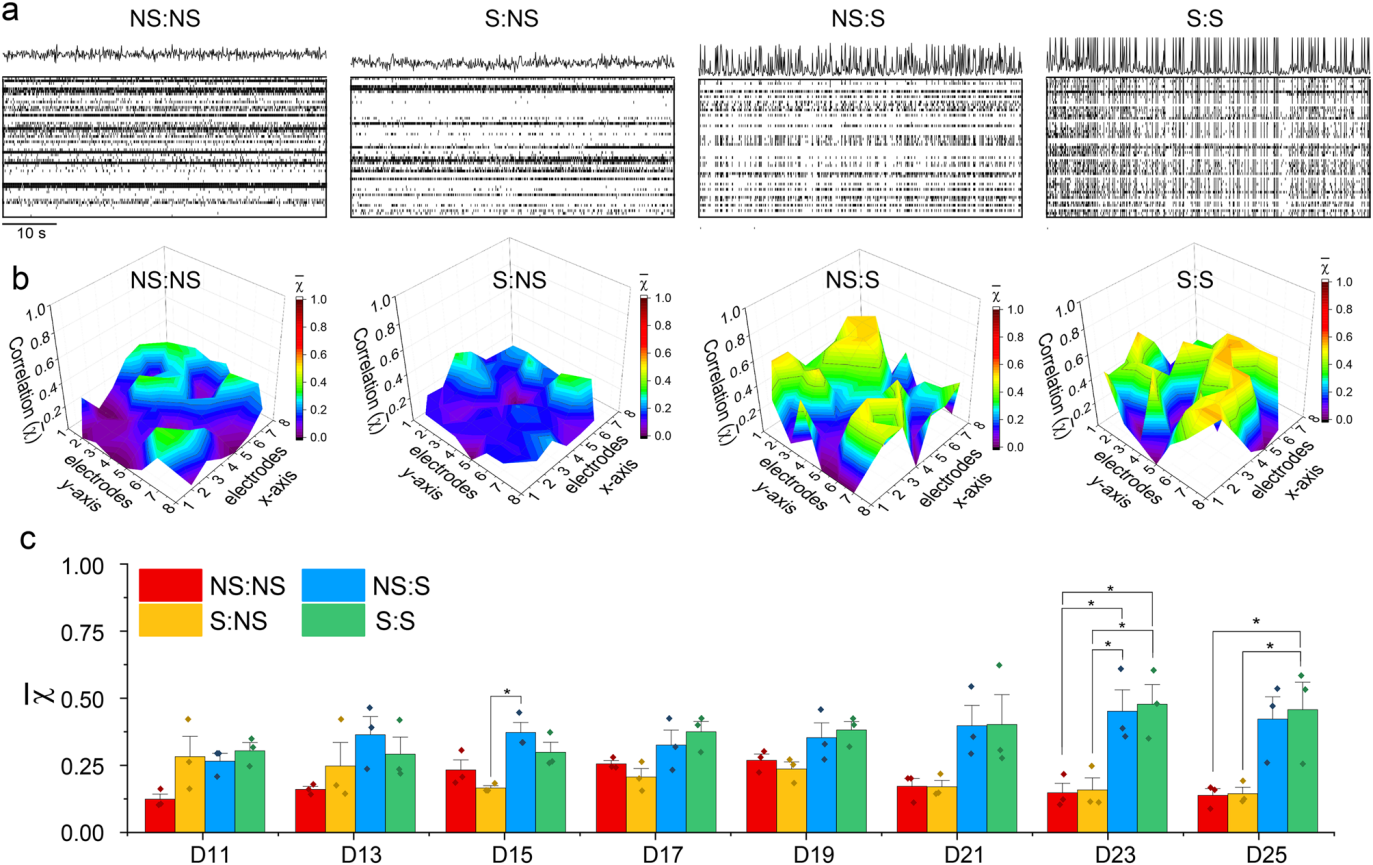
MEB network synchronicity is amplified by stimulation during neurogenesis and synaptogenesis. **a.** Representative raster plots of MEB cultures at D25 showing network synchrony by line plots of the sum of active electrodes for each time point. **b.** The average correlation value (χ) was calculated for active electrodes across time for an average value for each electrode, then mapped to their respective spatial position on the MEA array. **c.** Bar graphs representing the mean correlation value across the culture for the MEA cultures at the different days of recording. The correlation value for the culture was calculated using active electrodes during spontaneous time of each culture for each day of recording. Each point corresponds to the correlation value across electrodes for each MEA culture. (n = 3; error bar represents SEM, *p < 0.05, ANOVA with Tukey post-hoc test).

Network synchrony is a common parameter used to characterize a developing neural network, as it gives information on the network’s plasticity and connectivity. Various studies have successfully shown that the presence of chronic stimulation results in improved network synchrony^36–38^. In our study, we wanted to observe the long-term effects of stimulation regimens on the network synchrony and determine if these effects were amplified or shifted when the training regimen during neurogenesis was extended during synaptogenesis. From the raster plots of the spontaneous activity recorded at D21, the increased level of synchronous activity was notable between NS:S and S:S samples versus S:NS and NS:NS (Fig. 4a). This can be appreciated by the peaks above the raster plots, which correspond to a summation of the activity across all electrodes, where synchronous networks would result in discrete peaks whereas in samples that lacked coordinated firing, the resulting line plot seemed to lack any peaks.

Similarity between electrode recordings was quantified with cross-correlation in order to quantify synchronous behavior. Values for the similarity across the network were obtained by calculating cross-correlation for all electrode combinations (Supplementary Fig. 3). For this analysis, only spontaneous recordings of active electrodes (electrodes detecting at least 10 spikes/min) were used to quantify the long-term effects of the training regimen on steady state synchrony. When average correlation values per electrodes were mapped to their position on the chip, NS:S and S:S samples showed high synchrony level ($\stackrel{-}{\chi }$ > 0.5) across the entire network for spontaneous recordings at D21 (Fig. 4b). This showed that synchronous behavior extended across the entire network and was markedly higher for networks that were stimulated during synaptogenesis.

Interestingly, when the network wide mean synchronicity was calculated for each recording day, a trend of higher synchrony was observed for samples that had been exposed to some form of training regimen (NS:S, S:NS or S:S) but no statistical significance was observed at D11 (ANOVA, n = 3; F(3,8) = 3.42, p = 0.073) and D13 (ANOVA, n = 3; F(3,8) = 1.77, p = 0.23). At D15, a statistically significant difference (ANOVA, n = 3; F(3,8) = 7.47, p = 0.010) was observed, with a post hoc Tukey test performed at p < 0.05 showing statistical significance between NS:S and S:NS $\stackrel{-}{\chi }$ values. Subsequently, while no statistical significance was observed for D17 (ANOVA, n = 3; F(3,8) = 3.88, p = 0.055), D19 (ANOVA, n = 3; F(3,8) = 3.58, p = 0.066) and D21 (ANOVA, n = 3; F(3,8) = 3.61, p = 0.065), a gradual trend was observed for the synchronicity of networks undergoing training during synaptogenesis (NS:S and S:S) being larger than their counterparts (NS:NS and S:NS). At D23, there was a statistically significant difference among the experimental groups (ANOVA, n = 3; F(3,8) = 8.73, p = 6.6E-3). Post hoc comparisons using Tukey test at p < 0.05 indicated that the $\stackrel{-}{\chi }$ value for NS:S and S:S were higher than both NS:NS and S:NS groups. This statistically significance was sustained for D25 (ANOVA, n = 3; F(3,8) = 6.46, p = 0.016), with the post hoc Tukey test showing significant difference between $\stackrel{-}{\chi }$ for S:S and $\stackrel{-}{\chi }$ for NS:NS as well as S:NS. (Fig. 4c).

#### Spectral density elucidates changes in steady state firing

**Figure 5:**
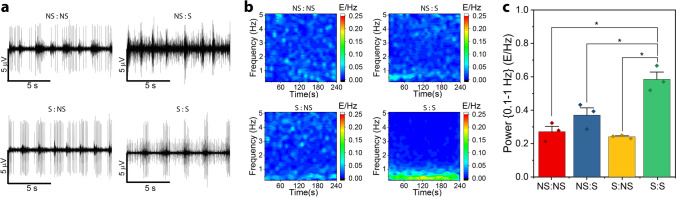
Stimulating training regimens modulates firing patterns in the frequency domain. **a.** Fifteen second representation of spontaneous voltage recording from NS:NS, NS:S, S:NS and S:S samples for D25. **b.** Smoothened (3 point moving average) and normalized (AUC) power spectra was calculated for contiguous 10 s windows across the 4 min of spontaneous recording NS:NS, NS:S, S:NS and S:S. Resulting matrices were averaged across samples. **c.** Bar graph for the sum of power spectral density magnitude from (**b**) across the spontaneous recording time between 0.1 Hz and 1 Hz (n = 3; error bar represents SEM, *p < 0.05, ANOVA with Tukey post-hoc test).

Conventionally, electrophysiological behavior is characterized by firing rate during set epochs and burst parameters (Supplementary Fig. 4). However, when analyzing these parameters during spontaneous firing, there was no discernable trend in the change of long-term firing rate or burst parameters between experimental groups. However, when observing the spike data during steady state of a more mature neural network (D25), there were deviations on how the spike firing clustered into bursts, despite the fact that no clear change in the number of spikes was observed (Fig. 5a). We accredited this seeming conflict between the quantitative and qualitative data to the selection method of the burst detection parameters (See Quantification and statistical analysis). In order to avoid arbitrariness in the selection of these parameters, we decided to characterize the data in the frequency domain. For this reason, we focused on characterizing spontaneous firing recorded on MEAs by comparing changes in the power spectrums of recorded signals calculated through Fourier transforms (Fig. 5b). To obtain spectral profiles, binned spike counts were divided into 10-s-long contiguous windows and transformed to the frequency domain, thus representing the power spectrum as a function of time (Fig. 5b). When initially calculating the power spectral density (PSD) and observing between the DC frequency and the Nyquist frequency, we noticed that most of the components appeared below 7 Hz for all samples. For this reason, we compared samples between 0.1 Hz (to remove DC component) and 5 Hz. Focusing between 0.1–5 Hz, all samples except S:S, showed frequency profiles of their respective firing patterns with components across the entire bandwidth of interest. This spontaneous heterogeneous firing patterns can be expected from these cultures formed from MEBs, as they are a super-network composed of individual networks from within each MEB. On the other hand, S:S samples show a clear change in their frequency profile, where most of the spectral power fell within 0.1-1 Hz.

Moreover, if the signal power is summed between the frequency range of 0.1-1 Hz, the training regimen pattern had a statistically significant effect at p < 0.05 on the power magnitude within this frequency interval (ANOVA, n = 3; F(3,8) = 20.15, p = 4.37E-4). Post hoc comparisons using Tukey test at p < 0.05 showed a statistically significant difference between power magnitude withing 0.1-1 Hz of samples non stimulated during synaptogenesis (NS:NS, S:NS) and samples stimulated throughout development (S:S) (Fig. 5c). Moreover, the post hoc Tukey test indicated a statistically significant difference between power spectra values between NS:S and S:S, implying that combined stimulation of both neurogenesis and synaptogenesis had an amplified effect on modulating the power spectra of the networks than just stimulation during synaptogenesis. This statistical significance was not observed in the mature networks (D25: ANOVA, n = 3; F(3,8) = 0.063, p = 0.98) if the power was summed for the whole frequency interval of interest (0.1-5 Hz) (Supplementary Fig. 5).

#### Neurogenetic stimulation changes the opto-response of MEB networks

**Figure 6:**
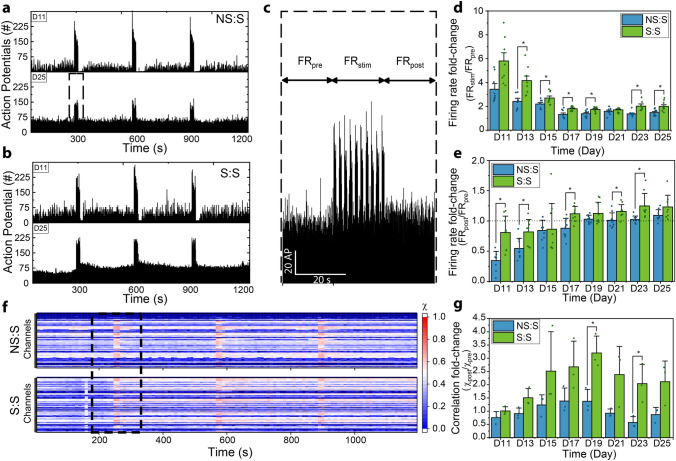
Stimulation during neurogenesis alters response to stimulation during network formation. Summed spike counts per each 100 ms for all active electrodes across the 20 min of recording were graphed for D11 and D25 for one representative sample from NS:S (**a**) and S:S (**b**). **c.** Zoom-in of **a** for 1 min, centered around the 20 s of stimulation at D25 for sample NS:S, the arrows represent the firing rate interval prior to stimulation (FR_pre_), the firing rate during stimulation (FR_stim_) and the firing rate after stimulation (FR_post_). **d.** Bar graphs showing the mean firing rate increase between Fr_stim_/Fr_pre_ for D11-D25. (n = 9; error bar represents SEM, *p < 0.05, ANOVA with Tukey post-hoc test)). **e.** Bar graphs showing the firing rate increase between Fr_post_/Fr_pre_ for D11-D25. (n = 9: error bar represents SEM, *p < 0.05, ANOVA with Tukey post-hoc test)). **f**. Raster plot of average correlation value for each electrode during 10 s bins across the entire recording time. **g**. Ratio of average correlation value prior to stimulation during recording and correlation value post stimulation (χ_post_/ χ_pre_). (n = 3; error bar represents SEM, *p < 0.05, ANOVA with Tukey post-hoc test).

Another aspect of consideration on the effect of training MEBs during neurogenesis was whether the early stage perturbation had some effects on how the later-stage network would respond to the same perturbation. To study this, we recorded responses to optogenetic stimulation from sets of samples that had not undergone the training regimen during neurogenesis (Fig. 6a) and compared them to those set that had undergone such regimen (Fig. 6b). Initial observation showed a difference between how the networks responded when stimulated early in the network development (D11) versus more mature networks (D25). For example, when early networks, which had a low spontaneous firing rate (D11) were stimulated, there would be a very notable evoked response during stimulation followed by a quiescent state, where the network would barely fire before returning to the baseline spontaneous firing rate. In contrast, more mature networks (D25), would still show an evoked response during stimulation but would automatically return to baseline firing rate right after stimulation ceased. What was interesting was that the quiescent time after stimulation for early S:S networks were notably shorter than those from the NS:S samples (Fig. 6a-b). Moreover, at D25, while NS:S samples would return to the same baseline firing rate right after stimulation stopped, S:S samples showed a transient change in firing rate for several seconds after the stimulation stopped (Fig. 6a-b).

To quantify this behavior, the evoked firing rate during stimulation (FR_stim_) and the post-response firing rate (FR_post_) were compared to the firing rate prior to stimulation (FR_pre_) for the three instances of stimulation within recording for each of the three MEA networks for both experimental groups (Fig. 6c). While the fold-change increase of firing rate FR_pre_ to FR_stim_ decreased with time for both NS:S (repeated measures ANOVA with Greenhouse–Geisser correction, n = 3; F(1.48, 11.83) = 14.79, p = 1.12E-3) and S:S (repeated measures ANOVA with Greenhouse–Geisser correction, n = 3; F(1.88, 15.02) = 11.02, p = 1.31E-3 (because more mature networks would have a higher baseline firing rate), when comparing the amount of evoked action potentials during stimulation (FR_stim_/FR_pre_), S:S samples seemed to respond more strongly to stimulation than NS:S samples (Fig. 6d). One-way ANOVA determined a statistically significant difference between NS:S and S:S FR_stim_/FR_pre_ values for D13 (n = 9; F(1, 16) = 5.55, p = 0.031), D15 (n = 9; F(1,16) = 5.90, p = 0.027), D17 (n = 9; F(1,16) = 11.30, p = 4E-3), D19 (n = 9; F(1,16) = 8.78, p = 9.2E-3), D23 (n = 9; F(1,16) = 10.81, p = 4.6E-3) and D25 (n = 9; F(1,16) = 9.94, p = 6.2E-3), while only showing a trend (not statistically significant) of higher S:S FR_stim_/FR_pre_ values for D11 (n = 9; F(1,16) = 4.48, p = 0.05) and D21 (n = 9; F(1,16) = 1.1, p = 0.31).

Additionally, the quiescent state response post-stimulation observed in early days (D11, D13 and D15), reflected itself in FR_post_ being less than FR_pre_, resulting in FR_post_/FR_pre_ < 1 for NS:S and S:S samples. We observed that this transient decrease in firing rate was statistically significantly shorter for the S:S samples than the NS:S for D11 (ANOVA, n = 9; F(1,16) = 19.95, p = 3.9E-4) and D13 (ANOVA, n = 9; F(1,16) = 9.49, p = 7.2E-3) (Fig. 6e). Repeated measured ANOVA indicated that FR_post_/FR_pre_ ratios increased for both NS:S (Greenhouse–Geisser corrected, n = 9; F(3.06, 24.48) = 36.92, p = 2.69E-9) and S:S (n = 9; F(7,56) = 5.66, p = 5.63E-5). Furthermore, at later days of network development, it was notable that FR_post_/FR_pre_ was ~ 1 for NS:S, meaning that the steady state firing rate was indistinguishable from that immediately following the termination of stimulation. On the other hand, S:S samples showed FR_post_/FR_pre_ values above 1 from D17 forward, indicating that the network would transiently increase in firing rate right after stimulation. One-way ANOVA showed that this increase between FR_post_/FR_pre_ values for S:S and NS:S was statistically significant for D17 (n = 9; F(1,16) = 12.19, p = 3E-3), D21 (n = 9; F(1,16) = 6.94, p = 0.018) and D23 (n = 9; F(1,16) = 9.91, p = 6.23E-3), while only showing a non-statistically significant trend for D19 (n = 9; F(1,16) = 2.16, p = 0.16) and D25 (n = 9; F(1,16) = 3.76, p = 0.071). It is relevant to mention that these effects were observed while there was no perceivable change in efficiency of the blue light to activate the ChR2 ion channels and evoke a response in the networks (Supplementary Fig. 6). These observations were corroborated by repeated measures ANOVA performed at p < 0.05, which showed no statistically significance change in efficiency (repeated measures ANOVA, n = 12; F(2,22) = 1.25, p = 0.31).

To further study how the training regimens affected network response, we also quantified the evoked response reflected in the network’s synchronicity for the initial stimulation done on the initial spontaneous interval of recording. For this purpose, raster-plots of the average values of cross-correlation (as calculated for the analysis in Fig. 4) were calculated using 10 s bins across the entire 20 min of recording (Fig. 6f). When quantifying the short term effect of stimulation during recording had on network synchronicity, by comparing $\stackrel{-}{\chi }$_post_ to $\stackrel{-}{\chi }$_pre_, a trend was observed where the presence of a training regimen during neurogenesis seemed to cause the correlation fold-change ($\stackrel{-}{\chi }$_post_/$\stackrel{-}{\chi }$_pre_) for S:S samples to be higher than NS:S samples. One-way ANOVA detected a statistically significant difference between $\stackrel{-}{\chi }$_post_/$\stackrel{-}{\chi }$_pre_ for S:S and NS:S for days D19 (n = 3; F(1,4) = 16.49, p = 0.015) and D23 (n = 3; F(1,4) = 11.12, p = 0.029) (Fig. 6g).

#### Changes evoked by stimulation during neurogenesis result in genetic changes

**Figure 7:**
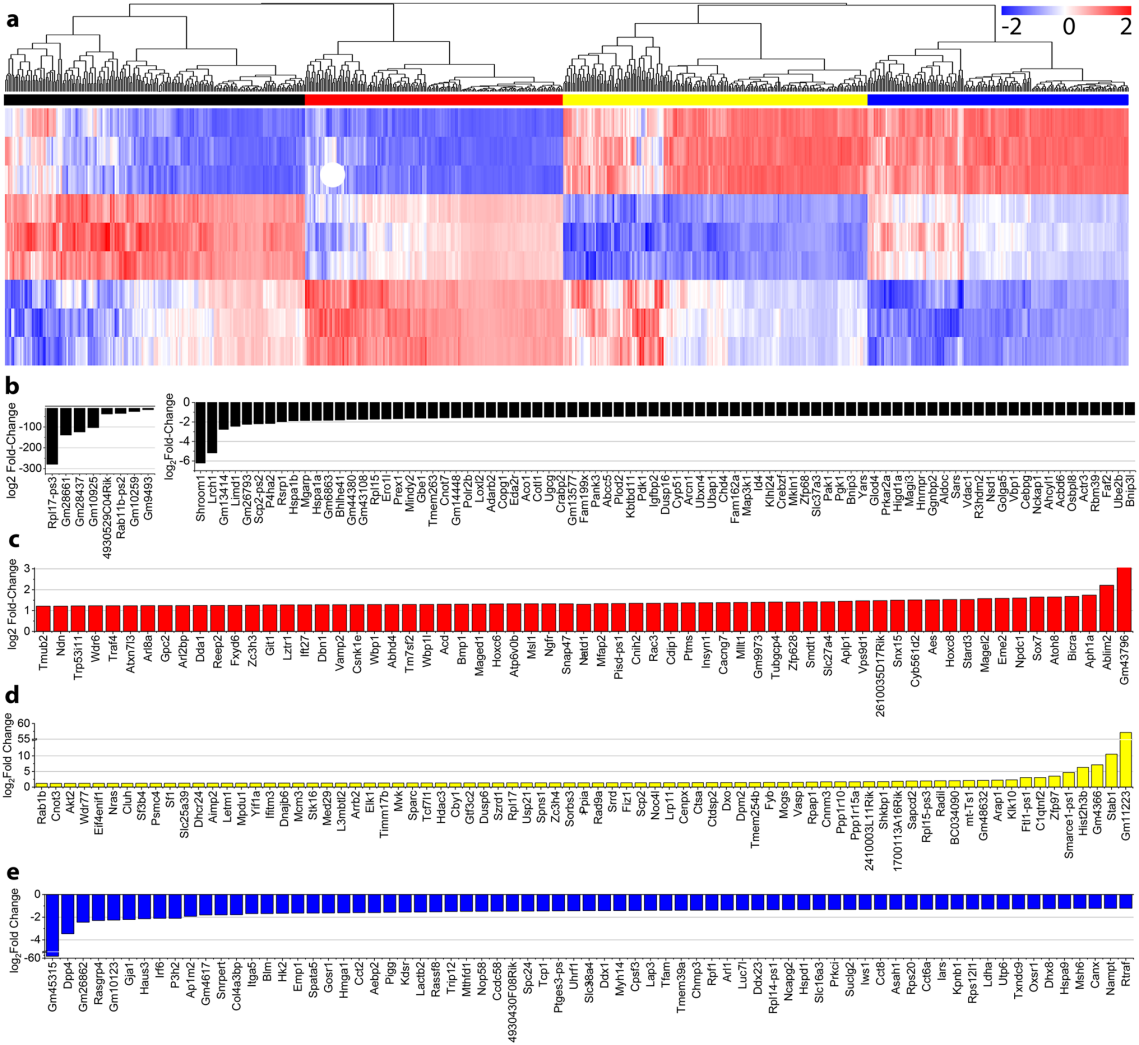
RNA Sequencing shows differential expression as a result of optical stimulation during neurogenesis. **a.** Heat map of standard deviation of differential expression for genes with p < 0.05 (n = 749). Genes were primarily clustered for: (1) genes that would overexpress during differentiation and underexpressed due to stimulation, (2) genes that would overexpress during control differentiation and overexpressed further due to stimulation, (3) genes that would underexpress during control differentiation and stimulation minimized that underexpression and (4) genes that would underexpress during control differentiation and stimulation amplified that underexpression. (first color column in order: black, red, yellow, blue). Significantly differentially regulated genes, with p < 0.0005 (n = 307) were extracted as column plots for: **b.** black, **c.** red, **d.** yellow and **e.** blue clusters.

Given the effects on neurite extension, presynaptic clustering, frequency profiles and network response to stimulation that were observed as a result of the presence of training regimens on MEBs during neurogenesis, we proceeded to determine genetic changes that could provide possible mechanistic explanations. Total messenger RNA sequencing was performed and analyzed for stimulated (S) and non-stimulated (NS) MEBs at D9, as well as EBs at D2. The differentially expressed genes in MEBs that underwent training regimens during neurogenesis were compared to those that did not, both with respect to the genetic expression of EBs sampled prior to differentiation (at D2). A total of 749 differentially expressed genes between S and NS with p < 0.05 were detected and clustered and color coded with respect to the differential expression of D2 (Fig. 7a). There were 200 genes that were upregulated during control differentiation, but this upregulation was lessened for samples that underwent training regimen (black bar), while the upregulation of 172 genes was amplified for those same samples (red bar). On the other hand, there were 202 genes whose downregulation was stagnated for samples with training regimen (yellow bar). For 173 genes, the control downregulation was further amplified after stimulation (blue bar). Something important to note was that this observed differential expression did not include changes in phenotype populations, matching the immunostaining observations (Supplementary Fig. 7). This indicated that training regimen during differentiation did not seem to noticeably disrupt the rate of phenotype specification or generation of the neural populations that generally result from the differentiation protocol (Table 1). This suggests that training regimens affected other functional pathways rather than altering the differentiation of populations. For further analysis, a more stringent threshold (p < 0.0005) was set to detect the most promising genes as key factors for the behavioral changes seen in stimulated MEB cultures. This threshold resulted in 97 differentially expressed genes for the black cluster (Fig. 7b), 63 differentially expressed genes for the red cluster (Fig. 7c), 77 differentially expressed genes for the yellow cluster (Fig. 7d) and 71 differentially expressed genes for the blue cluster (Fig. 7e). From this pool, a thorough literature study was used to identify gene targets that had been reported to be related to known neural development and function (Table 2, Supplementary Fig. 9).

**Table 1:** Expression comparisons for phenotypic gene targets.

Gene	Description	log.FC D2 v NS	FDR p	log.FC NS v S	FDR p
**ESC—Pluripotency**					
OCT ¾—(POU5F1)	POU domain, class 5, transcription factor 1	− 1,335.2	1.32E−08	− 1.01074	0.993303
NANOG	Nanog homeobox transcription factor	− 195.156	1.54E−08	1.291579	0.610174
SOX2	SRY (sex determining region Y)-box 2	− 1.1334	0.152156	− 1.15112	0.32166
**Neuronal Population**					
Neurod6	Neurogenic differentiation 2	78.42929	2.04E−09	1.298647	0.378425
Fox-3	RNA binding protein, fox-1 homolog 3	77.39941	3.59E−10	− 1.17408	0.501273
NEF	Neurofilament	92.5854	5.86E−13	− 1.02714	0.762375
**Astrocytes**					
Aqp4	Aquaporin 4	N/A	N/A	N/A	N/A
Gfap	Glial fibrillary acidic protein	27.17674	1.39E−07	− 1.14314	0.766754
Fgfr-3	Fibroblast growth factor receptor 3	59.96818	2.87E−10	1.029678	0.917007
**Oligodendrocytes**					
Mbp	Myelin basic protein	10.7271	8.42 E−09	− 1.00645	0.984504
Olig2	Oligodendrocyte transcription factor 2	− 1.24175	0.006191	− 1.21048	0.094616
Mog	Myelin oligodendrocyte glycoprotein	7.42182	5.11E−07	1.168882	0.60045
**Motor Neurons**					
ChAT	Choline acetyltransferase	46.15114	2.36E−06	1.55963	0.508302
MNX1	Motor neuron and pancreas homeobox 1	21.12197	5.35E−11	1.15419	0.234447
PAX6	Paired box 6	16.48545	2.91E−09	1.164541	0.454262
**Glutamatergic Neurons**					
Slc17a6	Solute carrier family 17 (sodium-dependent inorganic phosphate cotransporter), member 6	7.715048	4.04E−08	− 1.07919	0.747595
ADORA2A	Adenosine A2a receptor	5.181662	2.93E−05	− 1.10407	0.828501
Grina	Glutamate receptor, ionotropic, N-methyl D-aspartat E-associated protein 1 (glutamate binding)	3.733777	2.21E−09	1.173808	0.900906
**GABAergic Neurons**					
Slc6a1	Solute carrier family 6 (neurotransmitter transporter, GABA), member 1	68.94282	6.07E−11	1.07414	0.710842
GAD65	Glutamic acid decarboxylase 2	56.14012	3.24E−10	− 1.0758	0.764206
GABA	Gamma-aminobutyric acid receptor associated protein	2.496022	1.11E−07	1.239422	0.064079

**Table 2:** Significantly (p < 0.0005) differentially expressed genes reported in literature as regulators of neural development.

Gene	Description	Function	References
Npdc1	Neural proliferation, differentiation and control 1	Responsible for regulating differentiation. Upregulated in adult brains compared to young brains	Qu, X. et al.^38^, Evrard, C. et al.^39^, Galiana, E. et al.^40^, Sansal, I. et al.^41^
Crabp2	Cellular retinoic acid binding protein II	Upregulated in differentiated Motor Neurons, and downregulated in mature motor neurons	Boucherie, C. et al., Chaerkady, R. et al., Zhang, Q. et al
Snap47	Synaptosomal-associated protein, 47	Involved in unique fusion machinery for postsynaptic and presynaptic function	Münster-Wandowski, A. et al. Holt, M. et al. Arora, S. et al
Tubgcp4	Tubulin, gamma complex associated protein 4	Important in the nucleation and polar orientation of microtubules	Scheidecker, S. et al*.* Sánchez-Huertas, C. et al
Aplp1	Amyloid beta (A4) precursor-like protein 1	Supports maintenance of dendritic spines and basal synaptic transmission. High impact on synapse formation and synaptic plasticity. Is upregulated during synaptogenesis and is essential for proper synapse formation	Mayer, M. C. et al.^42^, Schilling, S. et al.^43^*,* Weyer, S. W. et al*.*^44^, Klevanski, M. et al.^45^*,* Kim, T.-W. et al.^46^
Cacng7	Calcium channel, voltag E-dependent, gamma subunit 7	Critical to Neural communication for Ca-dependent fusion of two secretory organelles: synaptic vesicles (SV) and neuropeptide-filled dense-core vesicles (DCV). Regulates the trafficking and gating properties of AMPA-selective glutamate receptors (AMPARs)	Yang, L. et al., Kato, A. S. et al
Cnih2	Cornichon family AMPA receptor auxiliary protein 2	Influences the efficacy of excitatory synaptic transmission. Slows synaptic transmission for reliable and successful transmission of a sugnal accros the synapse	Boudkkazi, S. et al., Boudkkazi, S. et al., Shi, Y. et al. Gu, X. et al
Insyn1	Inhibitory synaptic factor 1	Regulates postsynaptic inhibition and contributes to brain development	Gamlin, C. R. et al., Uezu, A. et al
Vamp2	Vesicle-associated membrane protein 2	Involved in the dockin and/or fusion of synaptic vesicles with the presynaptic membrane. It forms a distinct complex with synaptophysin	Russell, C. L. et al. Schwarz, T. L. Winkle, C. C. & Gupton, S. L. Winkle, C. C. & Gupton, S. L. Koo, S. J. et al
Reep2	Receptor accessory protein 2	Expressed in neuronal exocytotic tissue	Sjöstedt, E. et al. Esteves, T. et al. Hübner, C. A. & Kurth, I. Hurt, C. M. et al
Ngfr	Nerve growth factor receptor (TNFR superfamily, member 16)	Receptor for member of signaling pathway activating neurothrophins, p75NTR	Huang, E. J. & Reichardt, L. F. Barrett, G. L. & Bartlett, P. F
Nptx1	Neuronal pentraxin 1	Key factor in synapse loss and neurite damage	Omeis, I. A. et al., Abad, M. A. et al., Dodds, D. C. et al.,
Tuba1a	Tubulin, alpha 1A [	Key factor in axon and dendritic growth and network development	Belvindrah, R. et al. Aiken, J. et al

## Discussion

Engineering of neural circuits is a critical step for biomedicine as well as various neuroengineering efforts. This work aimed to expand upon previous findings on achieving long term modulation of a neural network’s behavior.^10,13,16,39–42^. Here, we showed a direct relation between changes in network function and development and training regimens implemented during neurogenesis and synaptogenesis. To our knowledge there has been no electrophysiological characterization of mESC-derived neural networks’ electrical response where a relationship between training during neurogenesis and synaptogenesis is examined. Furthermore these effects were correlated and supported by RNA-seq studies, where we showed that the training regimen affected targets related to network development, rather than affecting the differentiation of neural populations. In mESC-derived embryoid bodies, stimulation during neurogenesis has shown that the system enters a heightened state for neurite extension, which could be correlated to enhanced network formation. Furthermore, our study suggests that the presence of stimulation during neurogenesis has caused the system to be more sensitive and responsive to external stimulation after network formation, which could have implications on research studies where MEBs are integrated in in-vivo nerve tissues that have suffered injury. By implanting “trained” MEBs that have an enhanced responsiveness to stimulation, the therapeutic effects that have been observed could in turn be enhanced as well.^3^

Furthermore, spectral analysis proved to be a useful tool in order to quantify changes in network behavior that might be missed by simply comparing firing rates. While firing rate did not clearly reflect any alteration to the steady state, transforming the electrical signal to the frequency domain served as an informative descriptor of alterations in the network dynamics as a result of the training regimens. While spectral analysis has already been proven to be effective in characterizing neural signals^43,44^, the results of this study further elucidates its ability to detect modulations in the activity of neural networks that might not come across clearly from temporal-based parameters. Using FFT to characterize the network’s firing patterns as a response to the training regimens during network formation complements various other network effects that have been observed in this study. For example, it has been shown that optogenetic stimulation is capable of causing a higher level of synchrony in cultures consisting of primarily excitatory neurons^37^. This would suggest a more structured firing in the form of a more consistent frequency profile across time, since synchronous behavior would be more viable in structured firing patterns versus a sporadically firing network. It is relevant to point out that the frequency profile in the S:S cultures while not significant on its own, confirms a modulation in the electrophysiological activity of this in-vitro model in response to the presence of the regimen across the development of the neural network. Furthermore, it serves as a proof of concept for the modulation of electrophysiological activity using this type of experimental setup. Following the results shown here, it would merit to explore further alterations to the network behavior using other analogous training regimens across the different developmental stages and using both spectral and temporal parameters to characterize what changes arise.

Another very notable observation regarding how the presence of training regimen implemented during neurogenesis resulted in long-term changes in the neural network was the difference of how the neural system responded to the stimulation during recording. During early culture on chip (D11-D15), stimulation would cause the system to enter a quiescent state following the forced state of rapid firing from the stimulation, yet this quiescent state was statistically significantly shortened for samples that had been stimulated during neurogenesis. Moreover, these distinctions extended beyond early stages of network formation, which also manifested as prolonged transient increases of network synchrony. This is relevant because, while tetanic stimulation has been correlated to potentiation, no change in baseline firing rate was observed. Therefore, if no long-term changes had been caused by training regimen during neurogenesis, there should have been no reason for the optogenetically evoked firing rate on chip to have shown any differences to the NS:S counterparts. This enhancement of response in samples that were subjected to training regimens during neurogenesis points to long-term systematic changes on how the system would respond to the stimulation during downstream stages of network development.

To further validate these observations, RNA sequencing was performed on MEBs showing that a large number of genes were found to have been differentially expressed as a result of the training regimen. To focus our findings, the search was narrowed to genes showing a fold change with p < 0.0005 and key genes related to neural function were selected. Upon observation of this narrowed list, we found that following training regimen during neurogenesis the expression of key developmental genes Npdc1 and Crabp2 more closely resembles that of more mature tissue. Other differentially expressed genes implied an improved development of key neural aspects such as axonal and dendritic growth (Tuba1a) and nucleation of microtubules (Tubgcp4). Furthermore, stimulated MEBs seemed to overexpress important factors related to vesicular signaling such as Snap47, Vamp2, Reep2, and more importantly Cacng7. Furthermore, various genes are directly related to improved synaptic function and plasticity. For example, upregulation of Aplp1, Cnih2 and Insyn1 would be targets of interest that could account for the structured firing patterns and the changes in the network responses to evoked stimulation. Considering the change in expression of these genes related to vesicle transport, synaptic transmission and neural development, the observations of shorter quiescent times post-stimulation for samples that underwent training regimens could suggest that the training regimen enhanced the network for a quicker recovery from synaptic fatigue, which is an activity-dependent temporary inability of neurons to transmit signals^45^. Moreover, it is worth noting that Cnih2 and Insyn1 are regulators of inhibitory signaling related to the slowing down of signal transmission to improve its reliability, yet a lot is unknown about how these targets affect larger scale pathways and neuronal function. While many factors responsible for regulating network plasticity have been discovered^11,36,46–48^, how these interconnect and result in a steady state firing pattern, and furthermore, how could this pattern be controllably shifted is still to be determined. By continuing plasticity studies which incorporate training with respect to developmental stages and coupling the changes observed in the electrophysiology behavior to changes in genetic expression, these questions might be better addressed. Moreover, expanding this study to obtain protocol-specific responses in gene expression would be crucial in understanding neural plasticity and learning mechanisms.

Nevertheless, the results presented in this study add a new dimension to neural circuit engineering, by taking advantage of the synergy between responses to stimulation during differentiation and the plasticity that emerges during network formation, and correlation to gene expression. This solidifies our initial hypothesis that implementing training regimens during early stages of neural circuit development should be considered as a critical factor for studying neural plastic phenomena. In order to further this study, improved training regimens and spatial stimulation patterns during neurogenesis and synaptogenesis could be used to further the characterization of the network response. Furthermore, this approach could be expanded to in-vivo systems in order to examine if these alterations in fact result in behavioral changes in a developing organism, and if the enhancement of mESC-derived MEBs have an effect regeneration studies by enhancement recovery of nerve tissue injury.

## Supplementary information

Supplementary information
