# Supplementary material for: Modulating electrophysiology of motor neural networks via optogenetic stimulation during neurogenesis and synaptogenesis
Source: Sci Rep. 2020 Jul 27;10:12460. doi: 10.1038/s41598-020-68988-y (PMC7385114; doi:10.1038/s41598-020-68988-y)
Supplement: Supplementary file 1 — Supplementary information [file 41598_2020_68988_MOESM1_ESM.pdf]

Supplementary Information for:

Modulating electrophysiology of motor neural networks via optogenetic stimulation during neurogenesis and synaptogenesis

Gelson J. Pagan-Diaz, Jenny Drnevich, Karla P. Ramos-Cruz, Richard Sam, Parijat Sengupta, Rashid Bashir

Corresponding Author
Prof. Rashid Bashir
Engineering Hall, University of Illinois, Urbana-Champaign
1308 W Green St, Urbana, IL 61801
(217) 333-2151
rbashir@illinois.edu

This PDF file includes:
Supplementary Fig. 1 to Supplementary Fig. 8

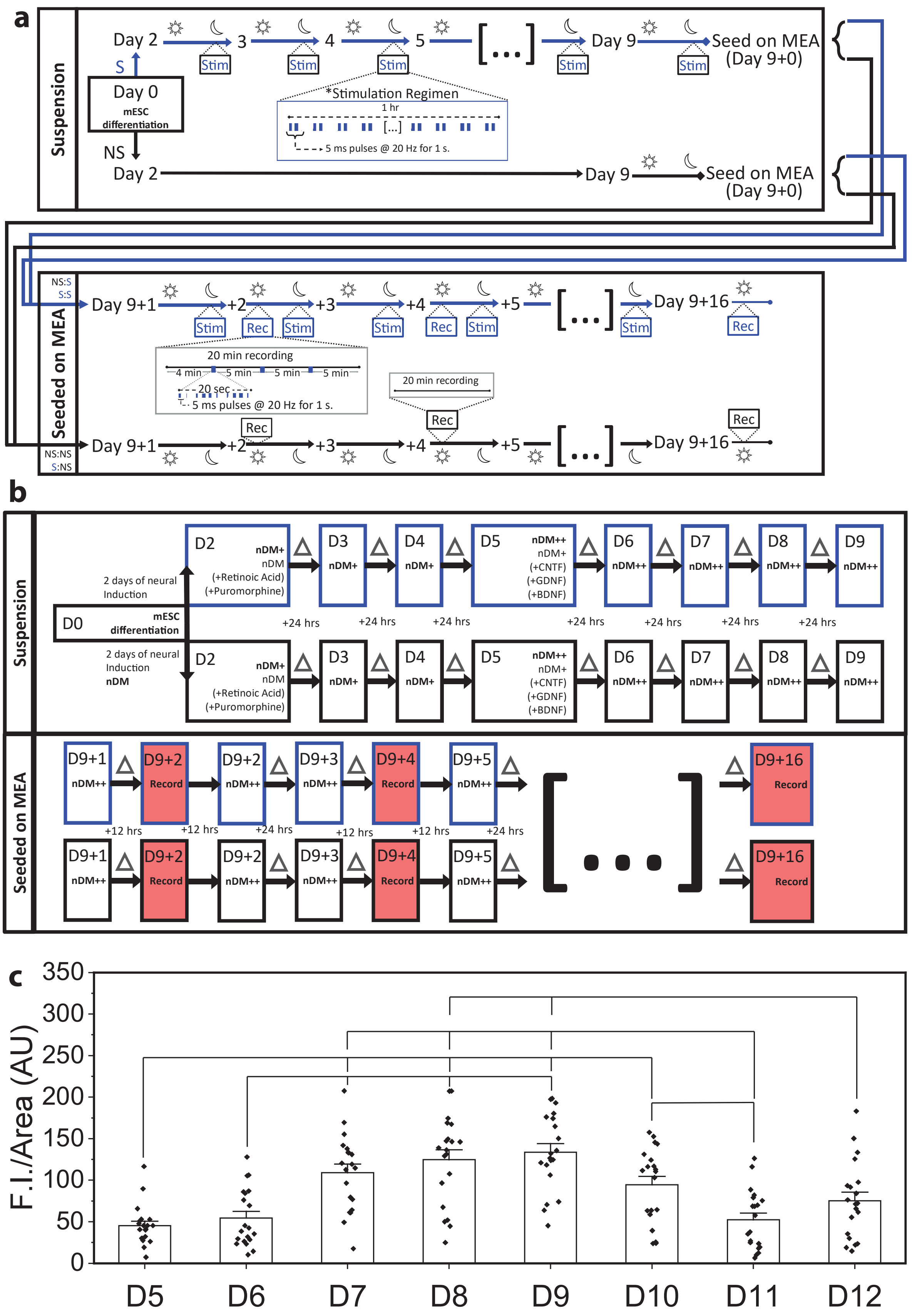

Supplementary Figure 1. a Stimulation regimen for differentiating and on chip samples. **b** Feeding regimen for samples **c** Bar graphs showing the mean HB9::GFP fluorescence during neurogenesis, normalized by the area of the embryoid bodies (n=20; error bar represents SEM). (Significance $p<0.05$; ANOVA with Tukey Post-hoc) ($F(7,152)=13.20$, $p=3.12E-13$)

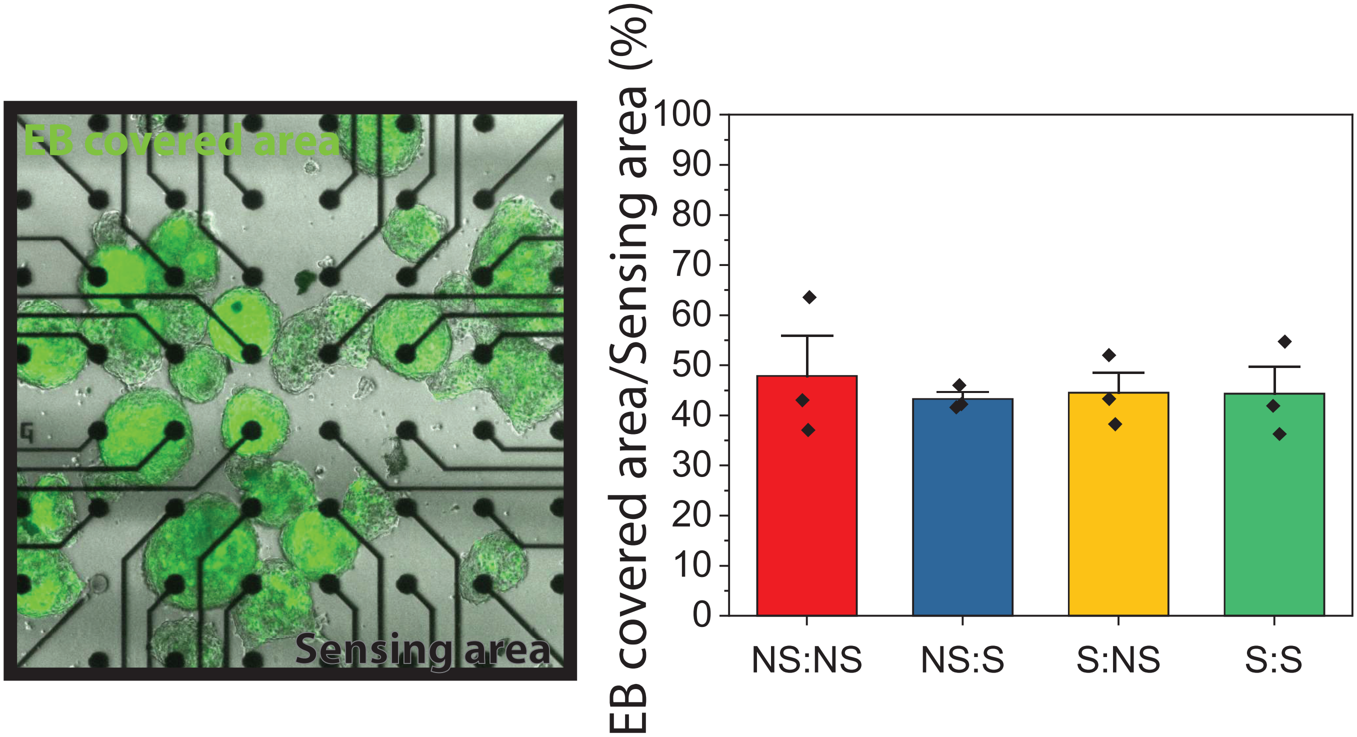

Supplementary Figure 2. Average coverage of MEBs on the MEA sensing area between groups (n=3, error bar represents SEM)(ANOVA; (F(3,8)=0.14, p=0.93)

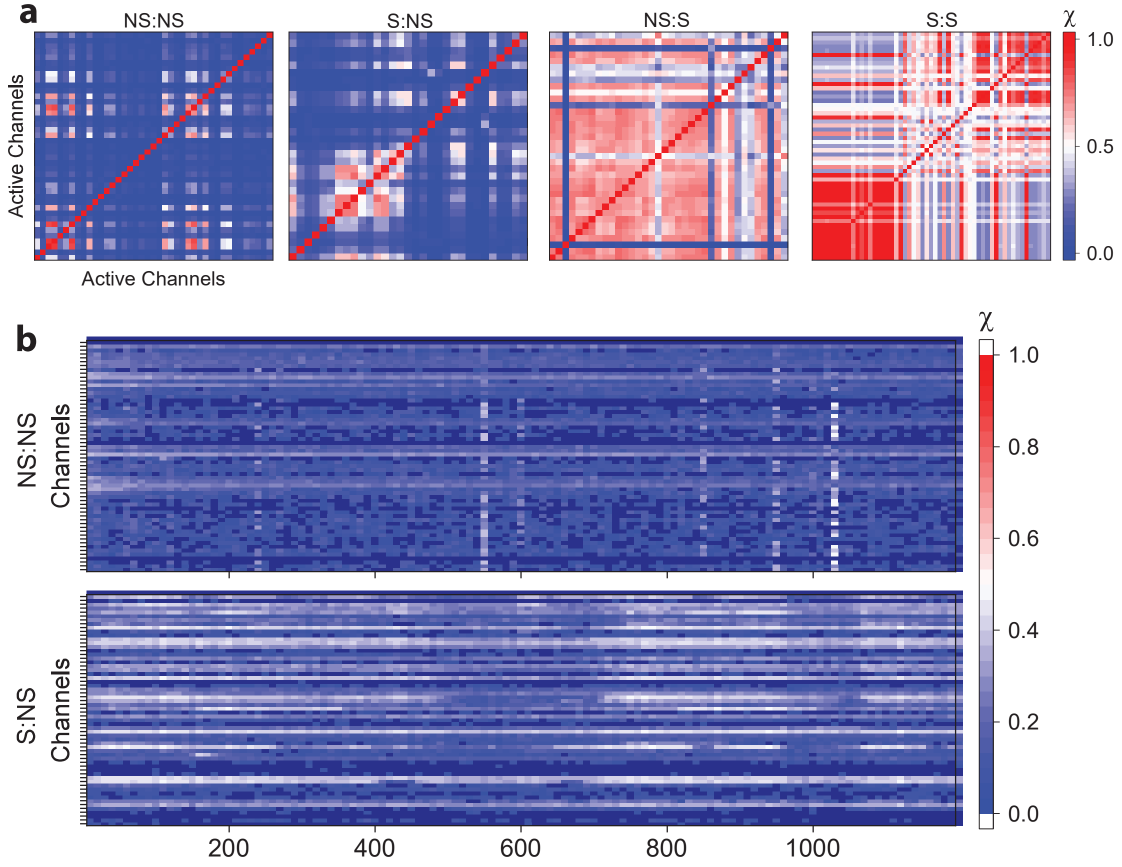

Supplementary Figure 3. a Cross-correlation matrices of mean correlation value (χ) across for the entire time of spontaneous activity for each active electrode to every other active electrode. Matrices were normalized to singularity for auto-correlation. **b** Raster plot of average correlation value for each electrodeduring 10 s bins across the entire recording time.

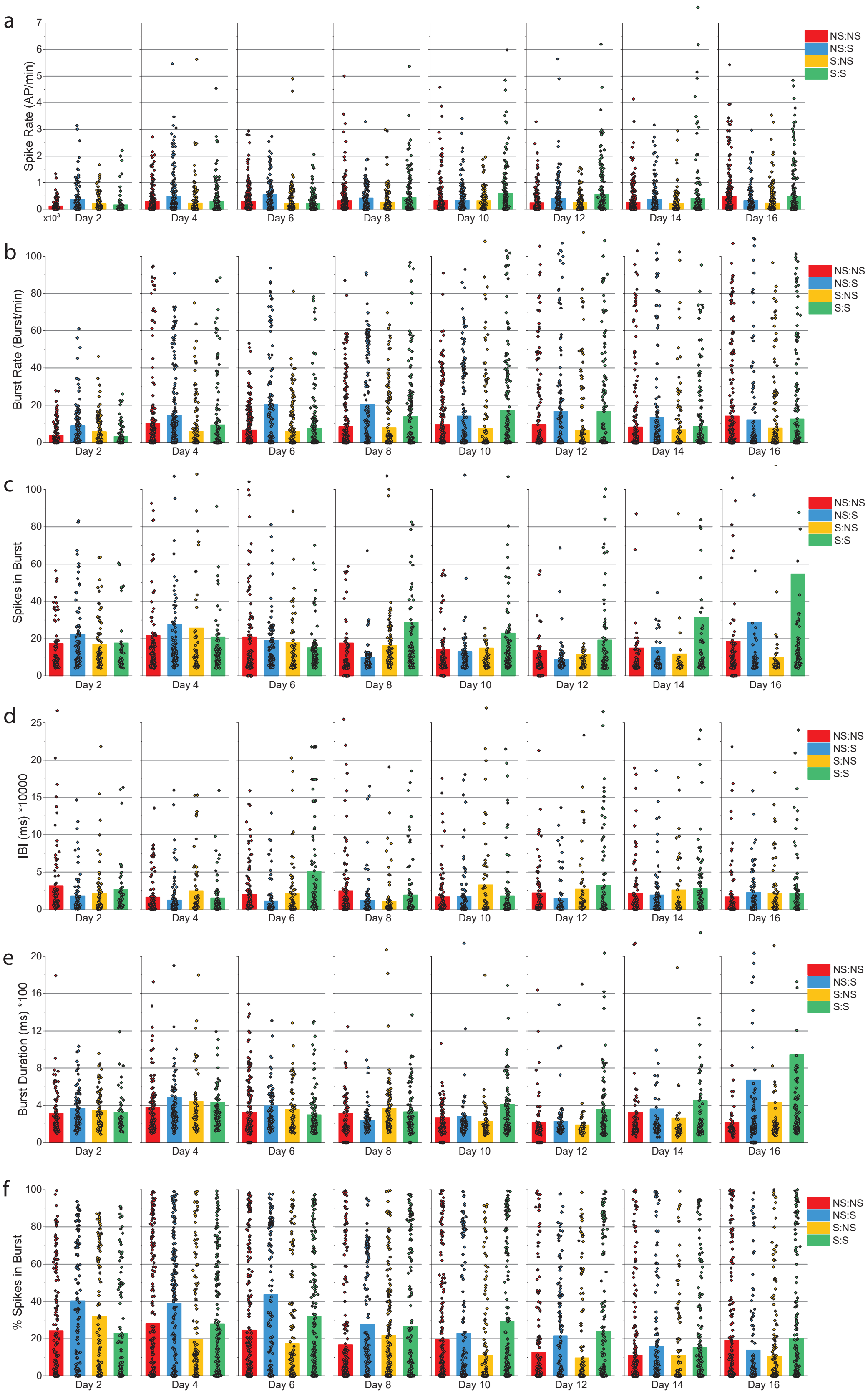

Supplementary Figure 4. Burst parameters for all active electrodes at each recording day for the spontaneous activity: **a** spike rate **b** burst rate **c** number of spikes in burst **d** intraburst interval **e** burst duration and **f** percentage of spikes found in bursts.

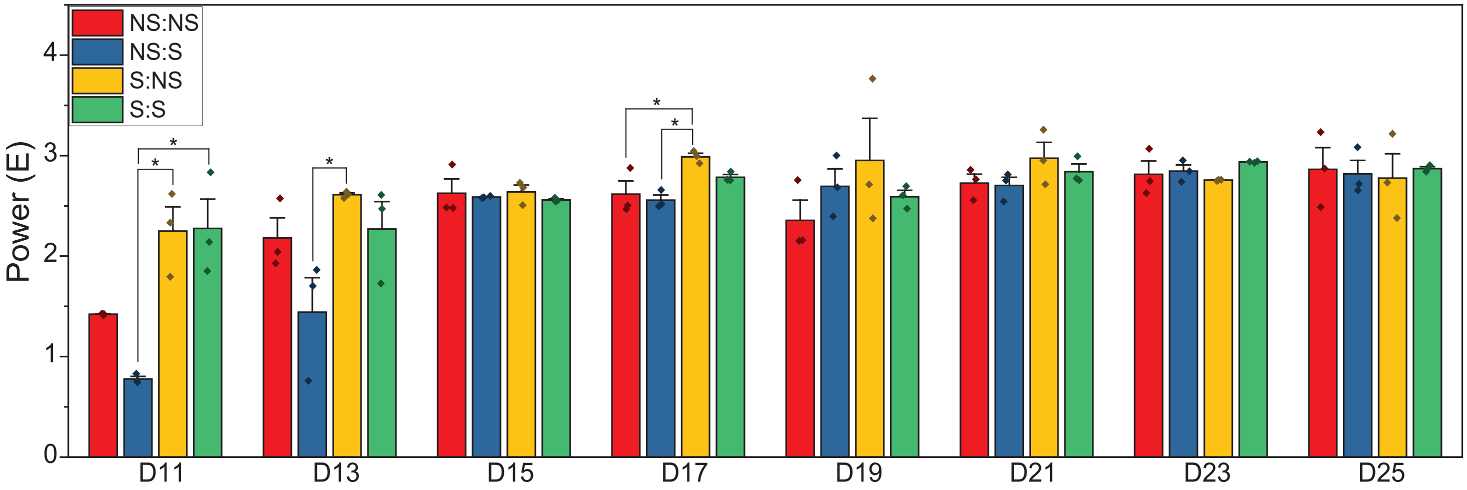

Supplementary Figure 5. Bar graphs representing the mean sum of total power spectra between 0.1-5 Hz between all four experimental groups for all recordings done during spontaneous activity (n=3; error bars represent SEM; *p<0.05; ANOVA with Tukey post-hoc test) (D11: F(3,8)=14.42, p=1.37E-3; D13: F(3,8)= 4.16, p=0.047; D15: F(3,8)=0.215, p=0.88; D17: F(3,8)=6.85, p=0.013; D19: F(3,8)=0.98, p=0.45; D21: F(3,8)=1.37, p=0.32; D23: F(3,8)=1.09, p=0.41; D25: F(3,8)=0.063, p=0.98)

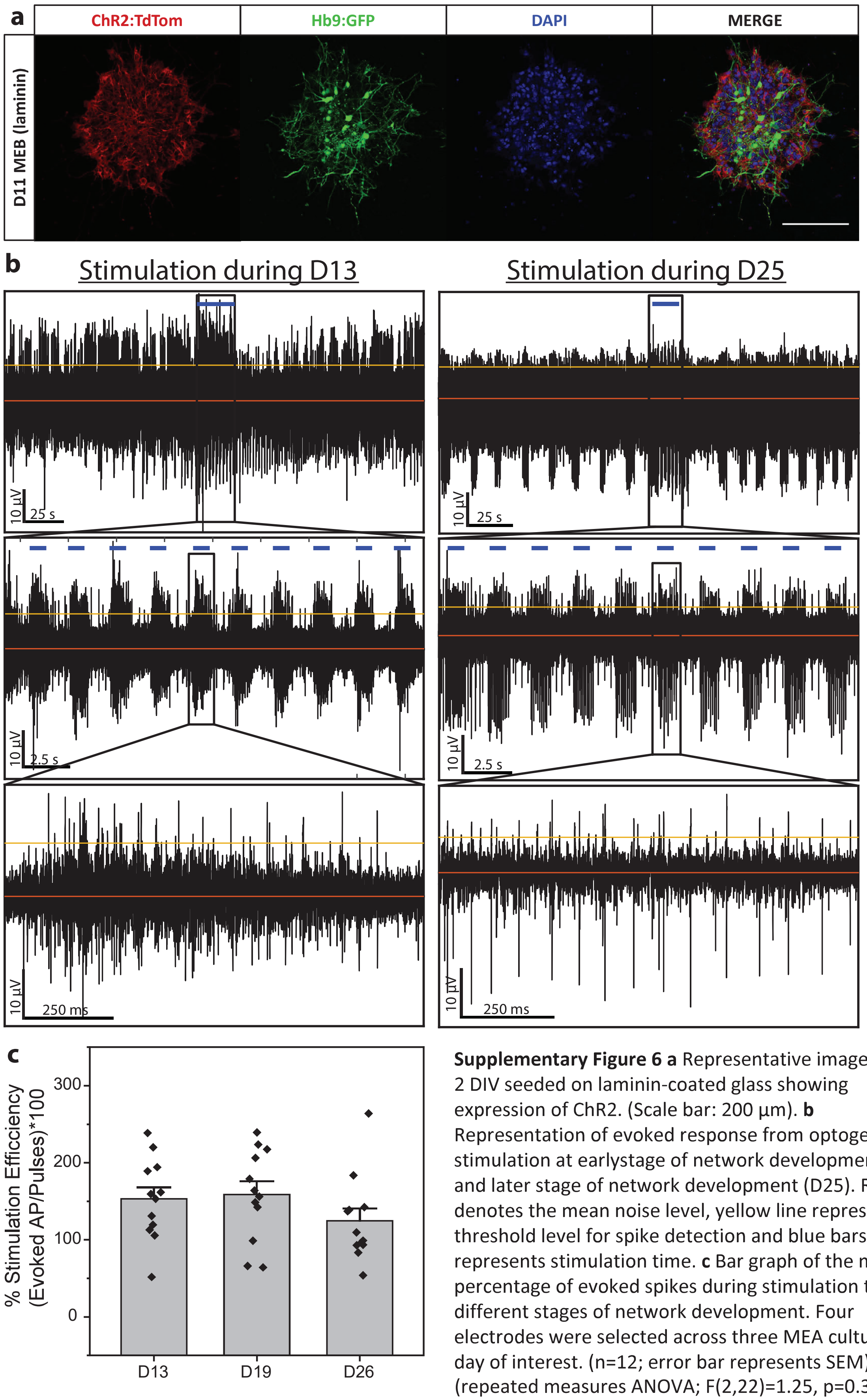

Supplementary Figure 6 a Representative image of MEB 2 DIV seeded on laminin-coated glass showing expression of ChR2. (Scale bar: 200 µm). **b** Representation of evoked response from optogenetic stimulation at earlystage of network development (D13) and later stage of network development (D25). Red line denotes the mean noise level, yellow line represents threshold level for spike detection and blue bars represents stimulation time. **c** Bar graph of the mean percentage of evoked spikes during stimulation times at different stages of network development. Four electrodes were selected across three MEA cultures per day of interest. (n=12; error bar represents SEM). (repeated measures ANOVA; $F(2,22)=1.25$, $p=0.31$)

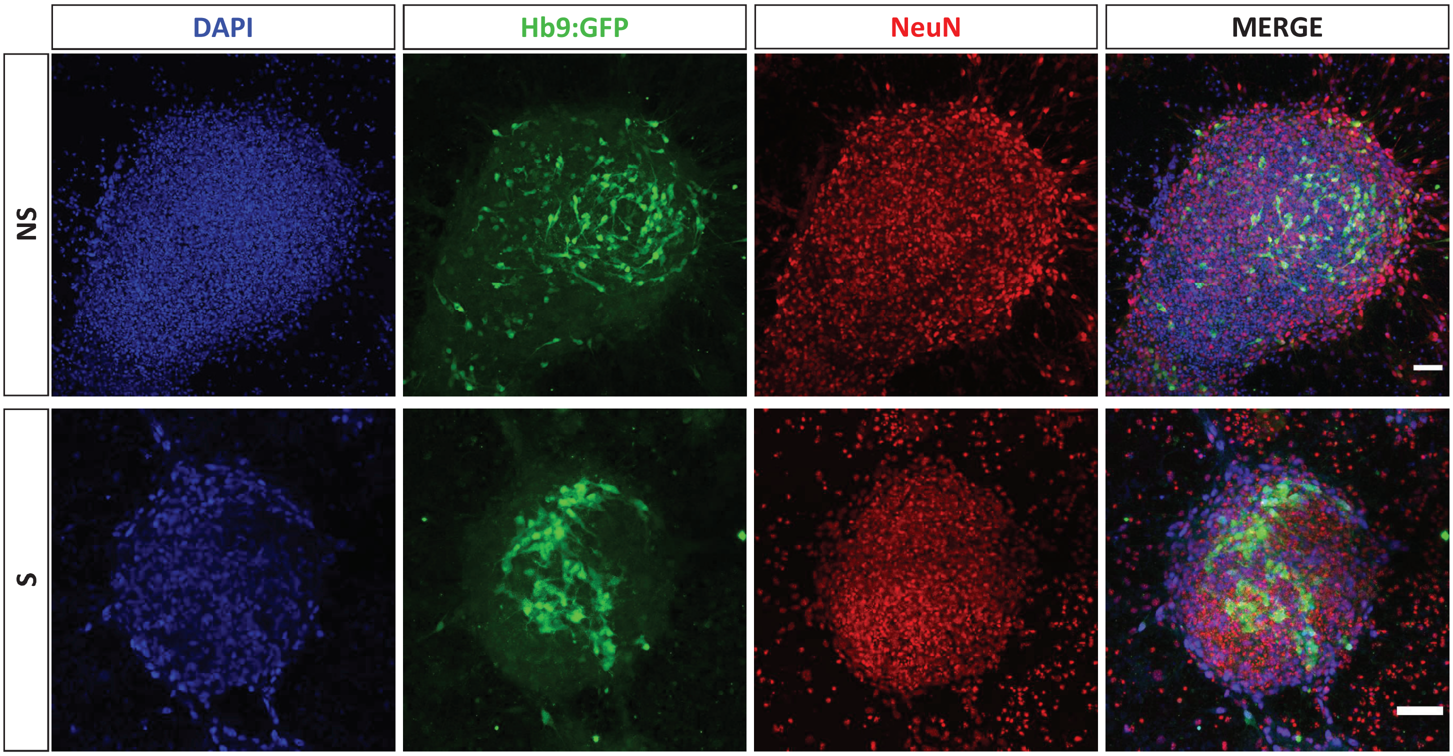

Supplementary Figure 7. Confocal images of stains for motor neuron expression for non-stimulated (NS) and stimulated (S) MEB at D9. (Scale bar: 50 µm)

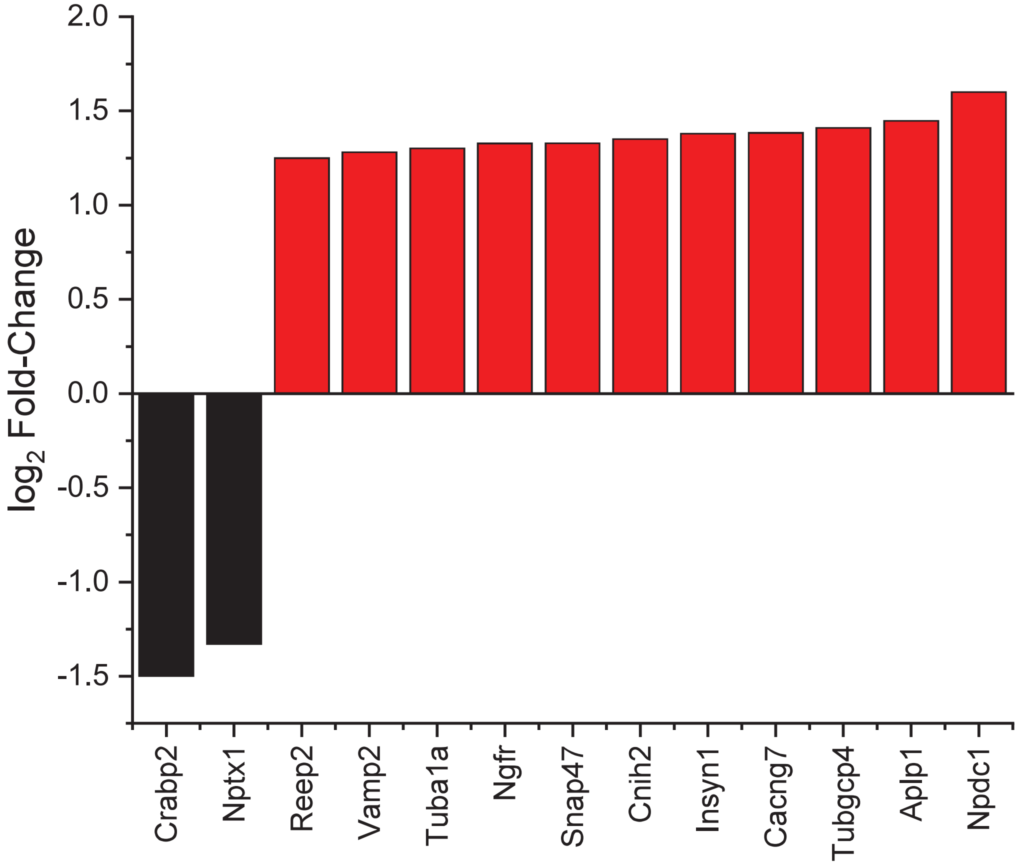

Supplementary Figure 8. Bar graphs showing the differentially expressed genes that satisfied the significance threshold ($p<0.0005$) and have been reported to be related to neural development and function.
